# Critical role of FPR1 in splenocyte migration into brain to worsen inflammation and ischemic brain injury in mice

**DOI:** 10.7150/thno.57218

**Published:** 2022-03-21

**Authors:** Jun Li, Mahendra D. Chordia, Yi Zhang, Hui Zong, Dongfeng Pan, Zhiyi Zuo

**Affiliations:** 1 Department of Anesthesiology, University of Virginia, Charlottesville, Virginia 22908, USA.; 2 Department of Radiology and Biomedical Imaging, University of Virginia, Charlottesville, VA, 22908, USA.; 3 Department of Microbiology, Immunology, and Cancer Biology, University of Virginia, Charlottesville, VA, USA.

**Keywords:** Brain imaging, brain ischemia, formyl peptide receptor 1, splenocyte migration, neuroinflammation, neuroprotection

## Abstract

**Background:** Splenocyte contribution to ischemic brain injury has been suggested. It is not known whether this effect is due to systemic action or direct influence in ischemic brain tissues. It is also not known how splenocytes migrate into the brain and worsen neurological outcome after brain ischemia. We determined the role of formyl peptide receptor 1 (FPR1), a receptor expressed in monocytes, in the migration of splenocytes into ischemic brain tissues and the contribution of these splenocytes to ischemic brain injury.

**Methods:** Mice with or without *fpr1* knockout were subjected to transient focal brain ischemia. The migration of splenocytes was assessed under *in vivo* and *in vitro* conditions.

**Results:** cFLFLF, a FPR1 antagonist, inhibited splenocyte migration into the brain and neuroinflammation after ischemic stroke. cFLFLF improved neurological outcome assessed 24 hours or 28 days after stroke. cFLFLF did not alter blood-brain barrier permeability in the ischemic brain. *fpr1*^-/-^ mice had an attenuated peripheral monocyte and neutrophil infiltration into the brain, a reduced proinflammatory cytokine level and an improved neurological outcome compared with wild-type mice after brain ischemia. cFLFLF did not affect the proinflammatory cytokine levels in the spleen and brain of *fpr1*^-/-^ mice after ischemic stroke.

**Conclusions:** These results suggest that FPR1 facilitates splenocyte migration into the brain and proinflammatory cytokine production to worsen neurological outcome after brain ischemia, indicating a direct effect of splenocytes on ischemic brain tissues. Our results support the notion that cFLFLF via blocking FPR1 signaling inhibits those pathological processes and is a potential agent for neuroprotection.

## Introduction

Ischemic stroke is a common cause of mortality and morbidity in the United States and the world 1. The underlying pathophysiological process is brain ischemic injury 2. Neuroinflammation is known to contribute to the injury after brain ischemia 2, 3. In this context, the spleen, an organ that contains a large number of immunoregulatory cells, may play a significant role in ischemic brain injury because splenectomy or irradiation of splenocytes reduces brain injury and neuroinflammation in rodents with ischemic stroke 4-7. Patients with stroke have decreased spleen volumes 8. Although there are some indications that splenocytes migrate into the ischemic brain 4-6, the molecular mechanism for this migration is not clear. Also, it is not clear whether the contribution of splenocytes to ischemic brain injury is due to their enhancement of systemic inflammation or direct effects on ischemic brain tissues.

The spleen stores many different types of cells including monocytes, macrophages and neutrophils. These cells express abundant formyl peptide receptors (FPRs) once they are activated 9. This process is initiated by pathogen-associated molecular patterns (PAMPs) or damage-associated molecular patterns (DAMPs) 10. There are at least three FPRs in mammals, FPR1, FPR2 and FPR3 11. FPR1 agonists are known to be a chemoattractant, promote chemotaxis, activate neutrophils and monocytes towards phagocytosis and induce a generation of reactive oxygen species and, thus, may be considered as proinflammatory and have been shown to cause injury 11-13. The agonist activity at FPR2 receptor by annexin-1 is shown to be anti-inflammatory by decreasing neutrophil-endothelium interaction, inhibiting monocyte chemotaxis and integrin-dependent cell adhesion, and down-regulating chemokine expression and function 11. The role of FPR3 remains unclear. FPRs have been shown to be involved in ischemia-reperfusion-induced injury in various organs and agents targeting FPRs have been shown to protect the heart, kidneys, lungs and intestines against ischemia-reperfusion 11. However, very limited information is available for the brain. One study has shown that AnxA1Ac2-26 reduces brain infarct size and inflammation after middle cerebral artery occlusion (MCAO) in mice. This effect may be attributed to the activation of FPR2 14. Activation of FPR2 also reduces leukocyte-endothelial interactions after a stroke 15. However, the role of FPR1 in ischemic brain injury is not known. The change of FPR1 expression in the monocytes, macrophages and neutrophils after brain ischemia has not been reported.

It has been shown that splenectomy reduces infiltrated monocytes, macrophages and neutrophils in the brain of mice after brain ischemia (reviewed in 16). We have synthesized the peptide cinnamoyl-phenylalanine-(D)leucine-phenylalanine-(D)leucine-phenylalanine (cFLFLF), a FPR1 specific antagonist 17. Based on the role of FPR1 in inflammation, we hypothesize that FPR1 plays a critical role in splenocyte migration into the brain and that cFLFLF provides neuroprotection by inhibiting migration of peripheral cells including splenocytes into the brain after brain ischemia. Mice with or without *fpr1* knockout were subjected to MCAO to address these hypotheses. Our results suggest that increased expression of FPR1 was necessary for the migration of splenocytes into the ischemic brain tissues after MCAO. cFLFLF inhibited this migration, attenuated neuroinflammation and improved neurological outcome after ischemic stroke in mice.

## Methods

The animal protocol was approved by the Institutional Animal Care and Use Committee of the University of Virginia (Charlottesville, VA). All animal experiments were carried out in accordance with the National Institutes of Health Guide for the Care and Use of Laboratory Animals (NIH publications number 80-23) revised in 2011 and reported according to the ARRIVE guidelines.

### Animal experimental protocol and cFLFLF administration

Eight-week-old male CD-1 mice weighing 28-32 g from Charles River Laboratories International, Inc. (Wilmington, MA) were housed under 12-hour light and 12-hour dark cycle with free access to food and water. Brain ischemia was a 1.5 h left MCAO. For the assessments that were performed within 24 h after brain ischemia, mice were randomly assigned to the following groups: 1) sham operated, 2) 0 h reperfusion, 3) 4 h reperfusion, 4) 24 h reperfusion, 5) 4 h reperfusion plus treatment of a single dose of 0.5 mg/kg cFLFLF, 6) 4 h reperfusion plus treatment of a single dose of 5.0 mg/kg cFLFLF, 7) 24 h reperfusion plus treatment of a single dose of 0.5 mg/kg cFLFLF, and 8) 24 h reperfusion plus treatment of a single dose of 5.0 mg/kg cFLFLF. The single dose of cFLFLF was administrated at the beginning of reperfusion through a tail vein in this experiment. To evaluate the protective time window, a single dose of 0.5 mg/kg cFLFLF was given at various times after the onset of brain reperfusion and the mice were randomly assigned to the following groups: 1) 24 h reperfusion without cFLFLF, 2) 24 h reperfusion plus cFLFLF given at the onset of reperfusion, 3) 24 h reperfusion plus cFLFLF given at 3 h after the onset of reperfusion, and 4) 24 h reperfusion plus cFLFLF given at 6 h after the onset of reperfusion. To evaluate whether cFLFLF improved long-term neurological outcome after brain ischemia, mice were randomly assigned to the following groups: 1) 28 d reperfusion, and 2) 28 d reperfusion plus treatment of three doses of 5.0 mg/kg cFLFLF. The first dose was given at the beginning of reperfusion through a tail vein and the following two doses were given at 24 h and 48 h after the first dose. The randomization was generated by a computer program. Neurological outcome including neurological deficit scores, performance on rotarod, and brain infarct volume as well as the spleen weight were evaluated at the corresponding times. Some mice were used for immunofluorescent staining.

Eighteen-month old male C57BL/6J mice weighing 33 to 37 g from the National Institute of Aging (Bethesda, MD) had left MCAO for 1 h. They were randomly assigned to the following groups: 1) 24 h reperfusion, and 2) 24 h reperfusion plus treatment of a single dose of 0.5 mg/kg cFLFLF given at the onset of reperfusion. Neurological outcome including neurological deficit scores, performance on rotarod, and brain infarct volume as well as the spleen weight were evaluated.

Cx3cr1CreERT2 (Stock No. 021160) and Rosa26-LSL-tdTomato reporter (Stock No. 007914) were from the Jackson Laboratory. Mice containing CreERT2 or tdTomato reporter were crossed. After genotyping verification, neonates containing both CreERT2 and tdTomato were injected with 4-hydroxytamoxifen (50 mg/kg/day) for five days continuously starting postnatal day 7. Twenty-eight days after the injection, all microglia had tdTomato (red fluorescence) but peripheral monocytes were colorless because of their fast turn-over rate. Afterwards, these mice were subjected to the 1.5 h MACO. At 4 h after the completion of MCAO, the immunofluorescent staining of cluster of differentiation 68 (CD68) in the ischemic brain cortex was determined.

*fpr1*^-/-^ mice were obtained as a gift from Dr. Phil Murphy at the National Institutes of Health (Bethesda, MD) and maintained in our laboratory. *fpr1*^-/-^ mice were bred on a C57BL/6J genetic background. Therefore, wide-type C57BL/6J mice from the Jackson Laboratory were used as the control animals. Eight-week old male *fpr1*^-/-^ mice and wild-type mice were subjected to 1.5 h MACO. Neurological outcome including neurological deficit scores, performance on rotarod and brain infarct volume as well as spleen weight were evaluated 24 h after MCAO. The levels of pro-inflammatory cytokines and mesangial cell-derived monocyte chemoattractant protein 1 (MCP1) in the spleen tissues and brain tissues harvested 4 h after MCAO were measured by ELISA and the level of phospho-p65 in the spleen was examined by Western blotting.

### MCAO

MCAO in mice was achieved by an intraluminal filament technique as we described previously 18, 19. In brief, surgery was performed under a dissecting surgical microscope. Mice were anesthetized with 1.8% isoflurane which is delivered continuously by a breathing loop with a facemask in pure oxygen. A small middle incision was made in the neck. The left common carotid artery (CCA), left external carotid artery (ECA) and left internal carotid artery (ICA) were isolated from the adjacent tissue. The distal portion of the left ECA was double-ligated with sutures, and the left ECA was cut between these two sutures. The left CCA and left ICA were temporarily clamped by using the microvascular clips. A small puncture was made on the cut end of the left ECA with a micro-scissor. A monofilament nylon suture (#1622, Beijing CiNongtech Co. Ltd., Beijing, China) with a rounded tip was advanced into the left internal carotid artery via the ECA until slight resistance was felt. After the microvascular clips were removed, the incision was infiltrated with 0.2% bupivacaine and the animal was allowed to awaken. Ninety minutes (for 8-week old mice) or sixty minutes (for 18-month old mice) after the onset of MCAO, the suture was withdrawn from the blood vessel under 1.8% isoflurane anesthesia to allow reperfusion. During the surgery to create MCAO, the rectal temperature of the mouse was maintained strictly at 37 ± 0.2 ºC by a thermostatic blanket. The blood flow in the MCA territory was monitored before and after the blood occlusion onset by a laser Doppler probe that was placed on the skull directly over the territory of the left MCA perfusion area. Animals were excluded from the experimental group if the cerebral blood flow did not decrease by 70% during occlusion or did not recover to 70% of baseline within 10 min after the start of reperfusion.

### Neurological outcomes assessment

Neurological deficit scores were evaluated in each group of mice after surgery based on an eight-point scale by a person blinded to the group assignment as we described before 18, 19. Mice were scored as follows: zero, no apparent deficits; one, failure to extend right forepaw fully; two, decreased grip of the right forelimb; three, spontaneous movement in all directions, contralateral circling only if pulled by the tail; four, circling or walking to the right; five, walking only if stimulated; six, unresponsiveness to stimulation and with depressed level of consciousness; and seven, dead.

Performance on rotarod was evaluated just before and at pre-determined times after the MCAO for the mice as we described before 18-20. Each group of mice was trained for three continuous days. Briefly, mice were placed on a rotarod with the speed accelerated from 4 to 40 rpm within 5 min. The latency and speed were recorded when a tested mouse fell off the rod. The speed-latency index, which was latency(s) × speed (rpm), was calculated. The ratio of this index obtained from before and after MCAO was calculated to reflect the change in coordinate function of each mouse.

After determination of the coordinate function, mice were euthanized by 6% isoflurane in a chamber. The assessment of infarct volumes in each group of mice within 24 h after the MCAO was performed after 2,3,5-triphenyltetrazolium chloride (TTC) staining by an independent investigator as we described before 18, 19. Briefly, mice were euthanized by 5% isoflurane and perfused transcardially with normal saline. Brains were harvested and sectioned into 2-mm-thick slices (usually 6 slices in total) over the entire brain. The slices were incubated in a 2% TTC solution in phosphate-buffered saline at 37 ºC for 10-20 min. The infarct area and the areas of the left and right cerebral hemispheres in each brain slice were qualified using Image J version 1.60 (National Institutes of Health). Corrected brain infarct volume in percentage = [right hemisphere volume-(left hemisphere volume - left infarct volume)] ×100/right hemisphere volume.

To evaluate infarct volume 28 days after brain ischemia, mice were euthanized by 6% isoflurane in a chamber and then transcardially perfused with 30 ml saline. Brains were removed and stored in 4% phosphate-buffered paraformaldehyde for 12 h at 4^o^C. Five-micrometer thick paraffin coronal sections were cut by using a microtome at 2-mm intervals (usually this is a total of 6 slices from one brain). Three sections were taken at the beginning and at the end of the 2-mm interval. The sections were stained with hematoxylin and eosin. The infarct areas in each brain section were quantified using the NIH Image J version 1.60. The sum of the infarct areas in the rostral and caudal sides of each brain slice was divided by 2 to get the average infarct area of the brain slice. The infarct volume of each brain slice was calculated by multiplying the average infarct area of the slice by the thickness of the slice (2 mm). The total infarct volume in the brain was the sum of infarct volumes of all slices from the brain. To account for differential shrinkage resulting from brain ischemia and tissue processing and to correct the individual difference in brain volumes, the percentage of infarct volume in the ipsilateral hemisphere volume was calculated. This determination was performed in a blinded fashion as it did with the evaluation of infarct brain volumes assessed by TTC staining.

Immediately after the MCAO, neurological deficit scores were evaluated and those mice with a neurological score less than 3 were excluded from the study due to the concern that MCAO was not that successful. All mice that were not excluded contributed data to the final analysis. If a mouse died before the end of the observation time point, the neurological deficit score and the ratio of speed-latency index of this mouse were 7 and 0, respectively, and were included for the final analysis. However, the mouse did not contribute infarct volume data for analysis. This method of data inclusion is the same as what we did before 19.

### Spleen weight

The spleen was harvested from each mouse and then weighed. The spleen weight was normalized to the body weight of that mouse.

### Preparation of ischemic brain homogenate (IBH) supernatant

At 0, 4 and 24 h after 1.5 h MCAO, brains were harvested and placed on ice. The ischemic infarct area was identified by its pale color with clear demarcation. The full thickness of the ischemic zone was then cut and suspended in phosphate buffered saline (PBS) (20 μl/mg tissue). After being homogenized with an electric homogenizer, the homogenate was centrifuged at 13,000 rpm for 20 min at 4 °C. The supernatant was acquired and the protein concentration of the IBH supernatant was determined by a BCA protein assay kit. The IBH was diluted to 5.0 mg/ml in PBS and stored at -20 °C before application. The normal brain homogenates were also prepared from sham surgery mice and stored at 5.0 mg/ml.

### Near infrared fluorescence (NIRF) imaging with Cy7-cFLFLF

We have previously described the synthesis and validation of cFLFLF as a synthetic-peptide ligand having affinity to FPR1 on polymorphonuclear leukocytes 21, 22. The synthesis of a Cy7-labeled cFLFLF (Cy7-cFLFLF) for NIRF imaging of inflammation was reported as well 23. In this study, we utilized freshly prepared cFLFLFK-PEG coupled with commercially available Cy7-NHS ester (Sigma-Aldrich, St. Louis, MO). CD-1 mice received a 100 μl tail vein injection of 2 nM Cy7-cFLFLF at the onset of reperfusion. The NIRF imaging was performed using an IVIS spectrum scanner (excitation 745 nm, emission 775 nm; PerkinElmer, Inc., Waltham, MA) under 1.2% isoflurane anesthesia 23, 24. The image acquisition, processing and semi-quantitative analysis were assisted by a live image software supplied by Caliper Life Sciences (Hopkinton, MA). To determine whether the accumulation of fluorescent dye in the ischemic brain regions was due to passive diffusion through impaired blood-brain barrier, CD-1 male mice received a 100 μl tail vein injection of 2 nM Cy7-cFLFLF or Cy7 24 h after MCAO. They were sacrificed by deep anesthesia and transcardially perfused with normal saline 2 h after the injection. Their brains were harvested immediately and imaged for NIRF signal.

### ^99m^Tc-cFLFLF distribution in organs after brain ischemia

Mice received ^99m^Tc-cFLFLF at ~37 MBq in 200 µl via a tail vein at the onset of reperfusion for mice that had 1.5 h MCAO plus 4 h reperfusion or 1.5 h after the onset of MCAO for mice that had 5.5 h MCAO. Their brain and spleen were harvested immediately at the end of experiments for counting their radioactivity *ex vivo* by a gamma counter (Wizard, Perkin-Elmer, Waltham, MA).

### C8-B4 cell culture and treatment

The C8-B4 cells (CRL-2540^TM^), a rodent microglial clone 25, 26, were obtained from American Type Culture Collection (ATCC, Manassas, VA) and cultured as we described previously 25, 26. Briefly, the cells were cultured in 25-cm^2^ culture flasks in Dulbecco's modified Eagle's medium supplemented with 10% fetal bovine serum (ATCC). They were maintained in a humidified atmosphere of 95% air/5% CO_2_ at 37 ºC in a cell culture incubator.

The cells were plated at a density of 5×10^5^ cells/well on 6-well culture plates. The cells were randomly assigned to four groups at 24 h after they were plated: IBH group, cFLFLF plus IBH group, *N*-formyl-methionyl-leucyl-phenylalanine (fMLP) group, and cFLFLF plus fMLP group. fMLP is the prototypical representative of the N-formylated oligopeptide family of chemotactic factors that act as a macrophage activator by the binding with FPR1 11. In the IBH group, the C8-B4 cells were incubated with 0.5 mg/well IBH on 6-well culture plates. In the cFLFLF plus IBH group, cFLFLF was added to make the final concentration of 0.1, 1.0 and 10 µM before 0.5 mg/well of IBH prepared from mice with 1.5 h plus 4 h reperfusion was introduced. In the fMLP group, C8-B4 cells were incubated with twelve final concentrations of fMLP ranging from 0 to 100 μM on 96-wells culture plates. In the cFLFLF plus fMLP group, 1.0 mM cFLFLF was added to the C8-B4 cells before the introduction of fMLP. The incubation with IBH or fMLP was for 4 h. They were then harvested for enzyme-linked immunosorbent assay (ELISA) of IL-1β and IL-6.

### Splenocytes isolation

Male CD-1 mice were euthanized by 6% isoflurane in a chamber and placed on a clean dissection board. After the animal was rinsed with 70% reagent alcohol, the abdominal cavity was opened and the spleen was removed. The excised spleen was sliced into small pieces in the petri dish with cell culture medium. The fragments of the spleen were placed onto a 40 μm cell strainer attached to a 50-ml conical tube and then pressed through the strainer with excess cell culture medium using the plunger end of a syringe. The cell suspension was centrifuged at 1,000 rpm for 5 min at room temperature. The supernatant was aspirated and the cell pellet was re-suspended in the 2.0 ml ammonium-chloride-potassium (ACK) lysis buffer and incubated for 15 min to remove the red blood cells. The cell suspension was centrifuged again at 1,000 rpm for 5 min at room temperature. The supernatant was discarded and the cells were re-suspended in cell culture medium. The isolated splenocytes were plated at a density of 5.0×10^6^ cells/well on 6-well culture plates and a density of 0.5×10^6^ cells/well on 96-well culture plates for ELISA of interleukin (IL)-1β and IL-6. The splenocytes were randomly assigned to four groups at 1 h after they were plated: fMLP group, cFLFLF plus fMLP group, IBH group and cFLFLF plus IBH group. The experimental process was the same as described above for the C8-B4 cells.

The isolated splenocytes from wild-type and *fpr1*^-/-^ mice were incubated with Cy5-cFLFLF in 6-well culture plates for 30 min or 4 h. After counterstaining with Hoechst 33342, these isolated splenocytes were mounted on a glass slide and imaged under a LSM710 microscopy system (ZEISS). Six non-overlapping fields from each slide were imaged. The number of pixels per image with intensity above a predetermined threshold level was considered as a positively stained area for Cy5 and quantified using Image J version 1.60. All quantitative analyses were performed in a blinded manner.

### Splenectomy and Cy5 labeled splenocyte injection

Splenectomy was performed as described before 27. Male CD-1 mice were anesthetized with 1.8% isoflurane delivered continuously by a breathing loop with a facemask in pure oxygen. A small midline abdominal incision was made and the peritoneal cavity was entered. The spleen was identified and brought to the incision. The hilum was clamped and ligated with the 6-0 suture and then the spleen was excised. The incision was closed in two layers using 5-0 Prolene. Two hours later, the mice were subjected to the 1.5 h MCAO. At the beginning of reperfusion, the cyanine 5 NHS ester (Cy5-NHS, catalog number: 5436; R&D Systems, Minneapolis, MN) labeled splenocytes were injected through the tail vein. To label the splenocytes, isolated splenocytes were incubated with Cy5-NHS ester for 30 min and then washed twice by PBS. Labeling on cells was confirmed via fluorescent microscopy. A total of 1.0 × 10^5^ of Cy5 labeled splenocytes were injected in each animal. Four hours after the MCAO, the mice were euthanized and the brain was harvested for detection of Cy5-labeled splenocytes in the ischemic cortex via confocal microscopy.

### Neutrophil isolation and treatment

Neutrophils were isolated from the preparations of splenocytes. Briefly and as described before 28**,** splenocyte suspensions prepared as described above were filtered through a 40 µm cell strainer and then were subjected to negative selection using the Easysep mouse neutrophil enrichment kit (catalog number: 19762; StemCell Technologies, Vancouver, BC). The isolated neutrophils were plated at a density of 0.1×10^6^ cells/well on 24-well culture plates and were randomly assigned to three groups at 1 h after they were plated: control group, fMLP group and cFLFLF plus fMLP group. In the fMLP group, neutrophils were incubated with 100 µM fMLP. In the cFLFLF plus fMLP group, cFLFLF was added to make the final concentration at 1.0 µM before the introduction of fMLP. The incubation with fMLP and cFLFLF was for 4 h. The level of IL-1β and IL-6 in the neutrophils after incubation were detected by ELISA assay.

### ELISA analysis

The protein levels of IL-1β, IL-6 and MCP1 were determined by using ELISA kits (catalog number: MLB00C for IL-1β, M6000B for IL-6; R&D Systems) according to the manufacturer's instruction and as described previously 29, 30. Briefly, the ischemic cerebral cortex of mice, spleen tissues of mice, C8-B4 cells, isolated splenocytes or isolated neutrophils in each study group were homogenized on ice in the RIPA buffer containing 25 mM Tris-HCl (pH 7.6), 150 mM NaCl, 1% sodium deoxycholate, and 0.1% sodium dodecyl sulfate (Thermo Scientific, Rockford, IL) as well as a protease inhibitor cocktail (10 mg/ml aprotinin, 5 mg/ml pepstatin, 5 mg/ml leupeptin, and 1 mM phenylmethanesulfonylfluoride) (Sigma-Aldrich). Homogenates were centrifuged at 13,000 g for 20 min at 4 °C, and the supernatant was then collected. BCA protein assay of the supernatant was performed for each sample. The levels of IL-1β, IL-6 and MCP1 in the supernatant were then measured. The amount of IL-1β, IL-6 and MCP1 in each sample was then normalized by total protein content of the sample.

### Immunofluorescent staining

The immunofluorescent labeling and quantification of the staining were performed as we have described before 25, 29. Briefly, mice were killed under deep anesthesia by transcardial perfusion with 4% paraformaldehyde in PBS. Brains were harvested and post-fixed in 4% paraformaldehyde in 0.1 M PBS at 4 °C for 24 h, then dehydrated and embedded in paraffin. Coronal 5-μm sections of the cerebral hemisphere were cut sequentially and mounted on microscope slides. Antigen retrieval with sodium citrate buffer (10 mM sodium citrate, 0.05% Tween 20, pH 6.0) was performed at 95-100 °C for 20 min. After being washed in Tris-buffered saline (TBS), sections were blocked in 10% donkey serum plus 1% bovine serum albumin in TBS containing 0.3% triton-X 100 for 2 h at room temperature and then incubated at 4 °C overnight with the following primary antibodies: mouse monoclonal anti-CD68 antibody (1:100; catalog number: ab955; Abcam, Cambridge, UK), rabbit monoclonal anti-TMEM119 antibody (1:50; catalog number: ab209064; Abcam), rat monoclonal anti-lymphocyte antigen 6 complex locus G6D (Ly6G) antibody (1:100; catalog number: ab210204; Abcam) or rabbit polyclonal anti-microtubule associated protein 2 (MAP2) antibody (1:1000; catalog number: ab32454; Abcam). Sections were rinsed in TBS with 0.1% Triton-X 100. The donkey anti-mouse IgG antibody conjugated with Alexa Fluor 488 (1:200; catalog number: A11055; Invitrogen, Waltham, MA) or donkey anti-rabbit IgG antibody conjugated with NL557 (1:200; catalog number: NL004; R&D Systems) were incubated with the sections for 1 h at room temperature in the dark. After being washed in TBS, sections were counterstained with Hoechst 33342 (Thermo Scientific), rinsed and mounted with Vectashield mounting medium (H-1000; Vector Laboratories, Burlingame, CA). Specificity of immune-labeling was confirmed by incubating sections with pre-immune rabbit IgG (1:250; catalog number: ab199376; Abcam) in place of primary antibody. Under these conditions, no detectable labeling was observed. Images of immunostaining were acquired with an LSM700 microscopy system (ZEISS). The quantification was performed as described previously 25. Briefly, for each mouse brain, 4 non-overlapping fields from each of six sequential ischemic cortex sections were imaged. The number of pixels per image with intensity above a predetermined threshold level was considered as a positively stained area for an interested marker and quantified using the Image J version 1.60. All quantitative analyses were performed in a blinded manner.

Cryostat sections were prepared from the brain of transgenic mice, from the brain of CD-1 mice received Cy5 splenocytes and from the spleen of *fpr1*^-/-^ and wild-type mice. Brains were harvested and fixed in 4% paraformaldehyde in PBS for 1 h at room temperature and then incubated in 30% sucrose overnight at 4 °C before being frozen in optimal cutting temperature compound. Coronal 20-μm sections were cut by using a cryostat and mounted on microscope slides. The staining procedure was the same as described above except that there was no antigen retrieval process. Rabbit polyclonal anti-phospho-nuclear factor (NF)-κB p65 antibody (1:1000; catalog number: ab86299; Abcam) was used as the primary antibody for the staining of spleen sections.

### Chemotaxis assay

The chemotaxis assay was performed by using the μ-slide chemotaxis 3D chamber (Ibidi, WI) in the following way. Two reservoirs are separated by a thin central connecting slit. The architecture and dimensions of this chamber generate a linear and stable concentration gradient by diffusing the chemotactic factor within the connecting slit. For isolated splenocytes, 5 μl of 3×10^5^ cells/ml in the culture medium were seeded into the connecting slit. Cell free culture medium (20 μl) was seeded into the left reservoir. Chemotactic factor (20 μl) was added into the right reservoir. Four groups were studied with the use of splenocytes freshly isolated from CD-1 wild-type mice in the first study: 1) 1:1 cell culture medium:PBS, 2) 2.5 mg/ml normal brain homogenates in 50% cell culture medium, 3) 2.5 mg/ml IBH in 50% cell culture medium, and 4) 2.5 mg/ml IBH in 50% cell culture medium on cFLFLF pre-treated splenocytes. As described above, IBH was prepared from CD-1 mouse brain tissues harvested 24 h after MCAO. For the cFLFLF treatment group, splenocytes were incubated with 1.0 mM cFLFLF for 1 h before being seeded into the connecting slit. In the second study, freshly isolated splenocytes from wild-type C57BL/6J mice or mice with *fpr1*^-/-^ were tested for their responses to IBH.

Cell migration was monitored under a light microscope (Nikon TS100, Japan). The image was captured every 30 seconds for over 30 min. A total of 60 images for each assay were converted to a video by Image J version 1.60. Cell number was counted for every minute in the first five minutes and every 5 min in the rest 25 min by Image J version 1.60. Quantitative data were presented as the ratio of the cell number at a time point versus the cell number at time zero and was analyzed in a blinded manner.

### Measurement of blood-brain barrier permeability

The blood-brain barrier permeability was tested by Evans blue dye method as previously described 31. Three groups of CD-1 mice were studied: 1) sham operated, 2) 24 h reperfusion, and 3) 24 h reperfusion plus 0.5 mg/kg cFLFLF treatment. Evans blue (2% in saline, 5 ml/kg; Sigma-Aldrich, St. Louis, MO) was injected via a tail vein. Mice were deeply anesthetized 1 h after injection and transcardially perfused with saline to remove the intravascular dye. The ischemic cortex of each mouse was harvested and weighed. These ischemic brain tissues were homogenized in 1 ml 50% trichloroacetic acid (catalog number: T4885; Sigma-Aldrich, St. Louis, MO), incubated overnight at 4 °C, and centrifuged at 13,000 rpm for 30 min. The amount of Evans blue in the supernatant was quantified by a spectrophotometer at 620 nm. The results were expressed as micrograms per gram of tissues.

### Western blotting

After transcardial perfusion with ice-cold normal saline, the ischemic cortex of CD-1 male mice and spleen of *fpr1*^-/-^ and C57BL/6J wild-type mice were micro-dissected and placed in ice-cold RIPA buffer supplemented with a 1% cocktail of protease and phosphatase inhibitors. Tissues were homogenized and centrifuged at 13,000 rpm for 10 min at 4 °C. The supernatant was collected. Its protein concentration was determined by using the BCA method. Aliquots of protein (30-40 μg per lane) were separated on a polyacrylamide gel and then blotted onto a polyvinylidene difluoride membrane. The membranes were blocked with Protein-Free T20 Blocking Buffer (catalog number: 37573, Thermo Scientific) and incubated with the following primary antibodies overnight at 4 °C: rabbit polyclonal anti-FPR1 antibody (1:1000; catalog number: ab203129; Abcam), mouse monoclonal anti-NF-κB p65 antibody (1:1000; catalog number: 6956; Cell Signaling, Danvers, MA), rabbit monoclonal anti-phospho-NF-κB p65 antibody (1:1000; catalog number: 3033; Cell Signaling), rabbit monoclonal anti-glyceraldehyde 3-phosphate dehydrogenase (GAPDH) antibody (1:1000; catalog number: ab181602; Abcam), rabbit monoclonal anti-Ly6G antibody (1:1000; catalog number: 87048; Cell Signaling), or rabbit polyclonal anti-α tubulin antibody (1:1000; catalog number: 2144S; Cell Signaling). Appropriate secondary antibodies were used. Band volumes of the target protein were normalized to those of α tubulin or GAPDH for non-phosphorylated proteins and to those of corresponding non-phosphorylated proteins for phosphorylated proteins. The result of experimental animals was normalized to the corresponding mean values of control animals.

### Statistical analysis

Parametric results in normal distribution are presented as mean ± S.E.M. (n ≥ 3). The data from chemotaxis assay and inflammatory cytokine results within the same group were tested by one-way repeated measures analysis of variance followed by Tukey test. The comparison of the chemotaxis assay and inflammatory cytokine results between groups were tested by two-way repeated measures analysis of variance followed by Tukey test. All other data were analyzed by t-test or one-way analysis of variance followed by the Tukey test if the data were normally distributed or by Mann-Whitney rank sum test or one-way analysis of variance on ranks followed by the Tukey test if the data were not normally distributed. Differences were considered significant at P < 0.05 based on two-tailed hypothesis testing. All statistical analyses were performed with SigmaStat (Systat Software, Point Richmond, CA).

## Results

### Cells binding with cFLFLF were increased in the brain and spleen after brain ischemia

**Figure 1:**
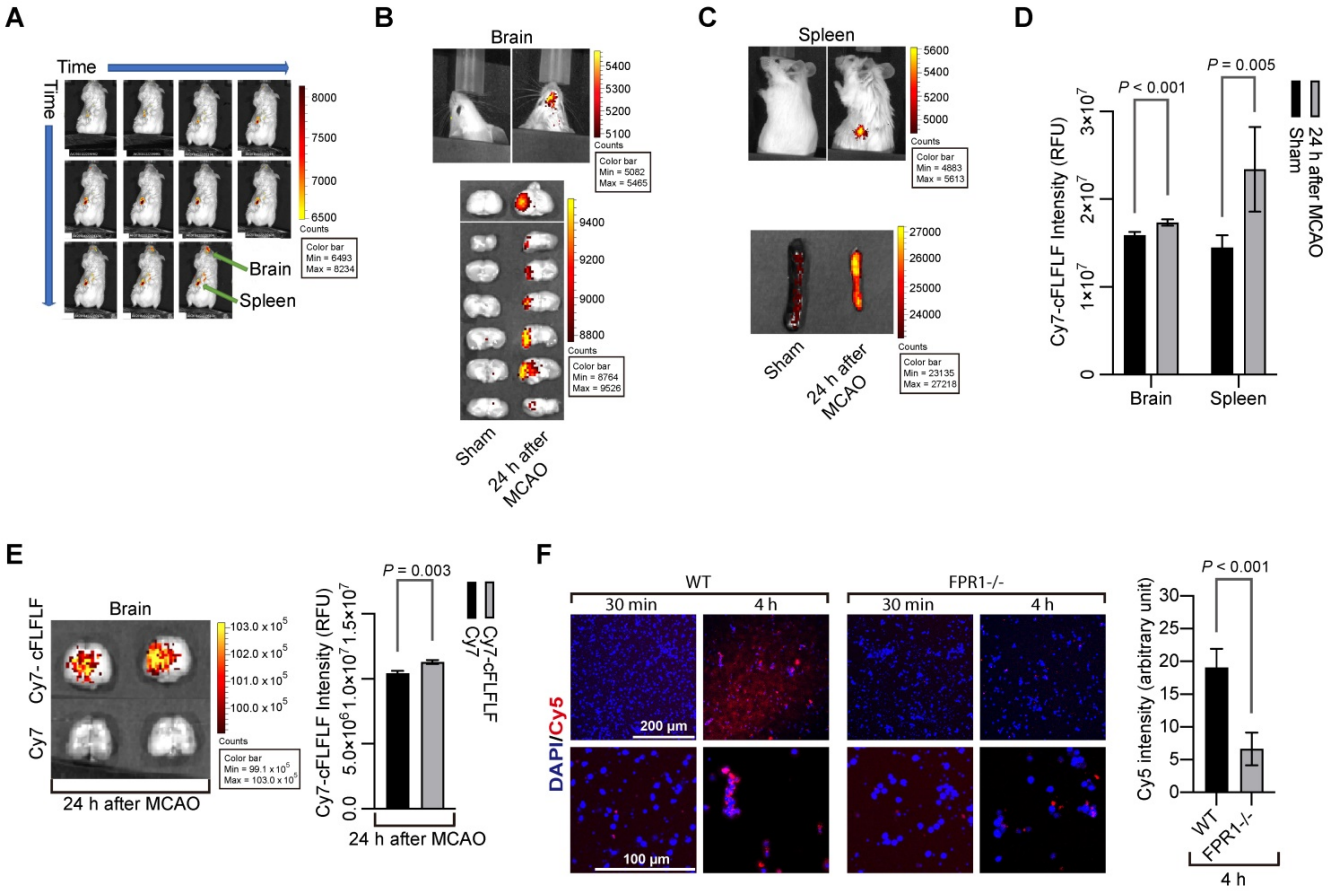
** cFLFLF via binding FPR1 to label cells in the brain and spleen after brain ischemia.** Mice received a 1.5 h left MCAO (panels A to E) and then various durations of reperfusion. **A:** representative whole-body images of NIRF after the mouse received intravenous injection of 100 µl of 2 nM Cy7-cFLFLF at 5 min before the reperfusion. The images were taken at 0, 1, 2, 3, 4, 6, 10, 15, 22, 32 and 47 min after the onset of reperfusion. **B:** representative brain images of NIRF after the mouse received intravenous injection of 200 µl of 2 nM Cy7-cFLFLF at 24 h after MCAO. Images were taken 2 h after the injection. **C:** representative spleen images of NIRF after the mouse received intravenous injection of 200 µl of 2 nM Cy7-cFLFLF at 24 h after MCAO. Images were taken 2 h after the injection. **D:** quantification of NIRF images in the brain and spleen. Results are mean ± S.E.M. (n = 8 for the brain, 6 for spleen). **E:** representative brain images and quantification of NIRF after receiving Cy7 or Cy7-cFLFLF. Mice received fluorescence dye and the brain was imaged as described for panel B. **F:** freshly isolated splenocytes from wild-type mice or mice with *fpr1*^-/-^ were incubated with Cy5 for 30 min or 4 h. Left two panels are representative images of these splenocytes. Right panel is the quantification of Cy5 intensity. Scale bar = 200 µm in the upper panels and = 100 µm in the lower panels. Results are mean ± S.E.M. (n = 6). T-test was used for panels D, E and F.

To determine whether cFLFLF could bind to cells, we prepared Cy7-cFLFLF (see supplemental materials for the synthesis of the probe). This probe (100 µl of 2 nM) was injected intravenously after 1.5 h MCAO. The mouse was scanned for NIRF at various time points after the MCAO in an *in vivo* imaging system (IVIS) spectrum scanner. As shown in Figure 1A, fluorescent signal appeared instantaneously upon the injection of the probe and then its intensity increased in the spleen. However, it took 22 min for the fluorescence to be seen in the brain. Mice had increased fluorescent intensity in the brain and spleen (Figure 1B-D). This increased fluorescent intensity was not due to passive diffusion of fluorescent dye through the impaired blood-brain barrier because Cy7-cFLFLF but not free Cy7 was accumulated in the ischemic tissues (Figure 1E). Cy7 is much smaller than Cy7-cFLFLF and shall be accumulated in those tissues if passive diffusion is the major mechanism for the accumulation of Cy7-cFLFLF in the ischemic brain tissues. To determine whether Cy7-cFLFLF bound to FPR1 expressing cells, freshly isolated splenocytes from mice with MCAO were incubated with Cy7-cFLFLF. Cy7-cFLFLF bound to cells upon incubation for 4 h and this binding was much less in the splenocytes isolated from *fpr1*^-/-^ mice (Figure 1F). These results suggest that brain ischemia has activated splenocytes and that there is an increase of FPR1-expressing cells in the brain.

### Peripheral monocytes and neutrophils including splenocytes migrated into ischemic brain tissues

**Figure 2:**
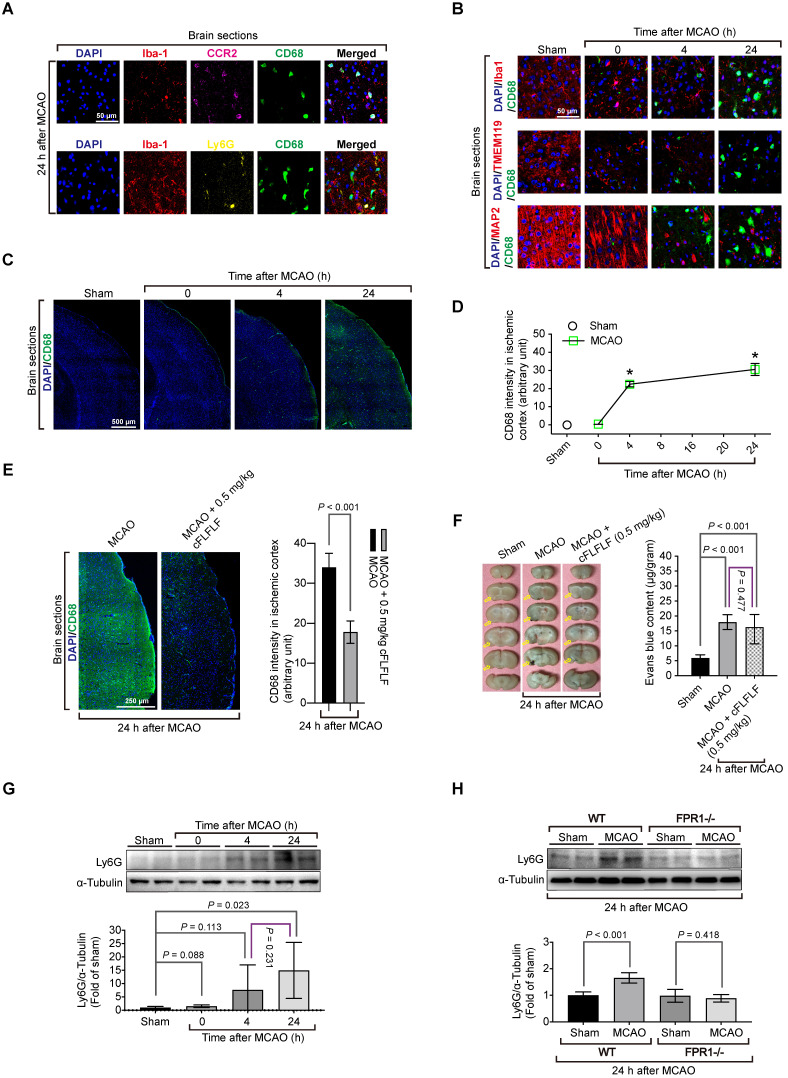
** Migration of peripheral cells into the ischemic brain and the role of FPR1 in this migration.** Wild-type mice were subjected to 1.5 h MCAO and then various durations of reperfusion. **A and B:** immunostaining of brain sections for various cell markers (Iba1 and TMEM119 for microglia, MAP2 for neurons, CD68 for monocytes, CCR2 for monocytes and Ly6G for neutrophils). Scale bar = 50 µm. **C:** images of tile scanning of brain sections for CD68 immunostaining in the ischemic cortex. Scale bar = 500 µm. **D:** quantification of CD68 immunostaining intensity in the brain. * P < 0.05 compared with sham operated animals. **E:** immunostaining of brain sections of mice treated with or without cFLFLF. Left panel is the images of tile scanning of ischemic cortex. Scale bar = 50 µm. Right panel is quantification of CD68 immunostaining intensity in the brain with or without the treatment of cFLFLF. **F:** blood-brain barrier permeability measured by Evans blue. Left panel: representative images of brain sections. Yellow arrows indicate ischemic brain area with Evans blue accumulation. Right panel: quantification of Evans blue assay. **G:** Ly6G abundance in the ischemic cerebral cortex of CD-1 mice. Top panel: representative Western blotting images. Bottom panel: quantitative data. **H:** Ly6G abundance in the ischemic cerebral cortex of C57BL/6J mice (wild-type: WT) and mice with *fpr1* knockout mice. Top panel: representative Western blotting images. Bottom panel: quantitative data. Results in panels D, E, F, G and H are mean ± S.E.M. (n = 6). One-way analysis of variance followed by the Tukey test was used for analyzing results in panels D, F and G and t test was used for analyzing data in panels E and H.

To determine which types of cells that bound to cFLFLF, we performed immunohistochemistry. There was a significant amount of cells positively stained with CD68, a marker for monocytes and macrophages 32, in the ischemic brain tissues but not in the normal brain tissues. CD68 positive cells were co-stained positively with C-C chemokine receptor type 2 (CCR2), another monocyte and macrophage marker 33 but were not co-stained with Ly6G, a marker for neutrophils 34 (Figure 2A). These CD68 positive cells were not co-stained positively with ionized calcium binding adaptor molecule 1 (Iba1) and transmembrane protein 119 (TMEM119), two specific microglial markers 25, 35, and were not co-stained with MAP2, a neuronal marker 36 (Figure 2B). The intensity of CD68 staining was increased with the time after the brain ischemia (Figure 2C-D) and was inhibited by 0.5 mg/kg cFLFLF given intravenously at the onset of reperfusion (Figure 2E). This inhibition may not be due to the decrease of passive permeability of CD68 positive cells through impaired blood-brain barrier because mice treated with cFLFLF had a degree of blood-brain barrier permeability increase similar to that of mice without cFLFLF after brain ischemia (Figure 2F). These results suggest that peripheral monocytes and macrophages that are CD68 positive may have infiltrated into the ischemic brain. Also, cells positively stained with Ly6G were not positively stained for Iba-1 in the ischemic brain tissues. The amount of Ly6G in these tissues was increased at 24 h after the MCAO (Figure 2G). This increase did not occur in *fpr1*^-/-^ mouse brain after MCAO (Figure 2H), suggesting a FPR1-dependent neutrophil migration into ischemic brain tissues.

**Figure 3:**
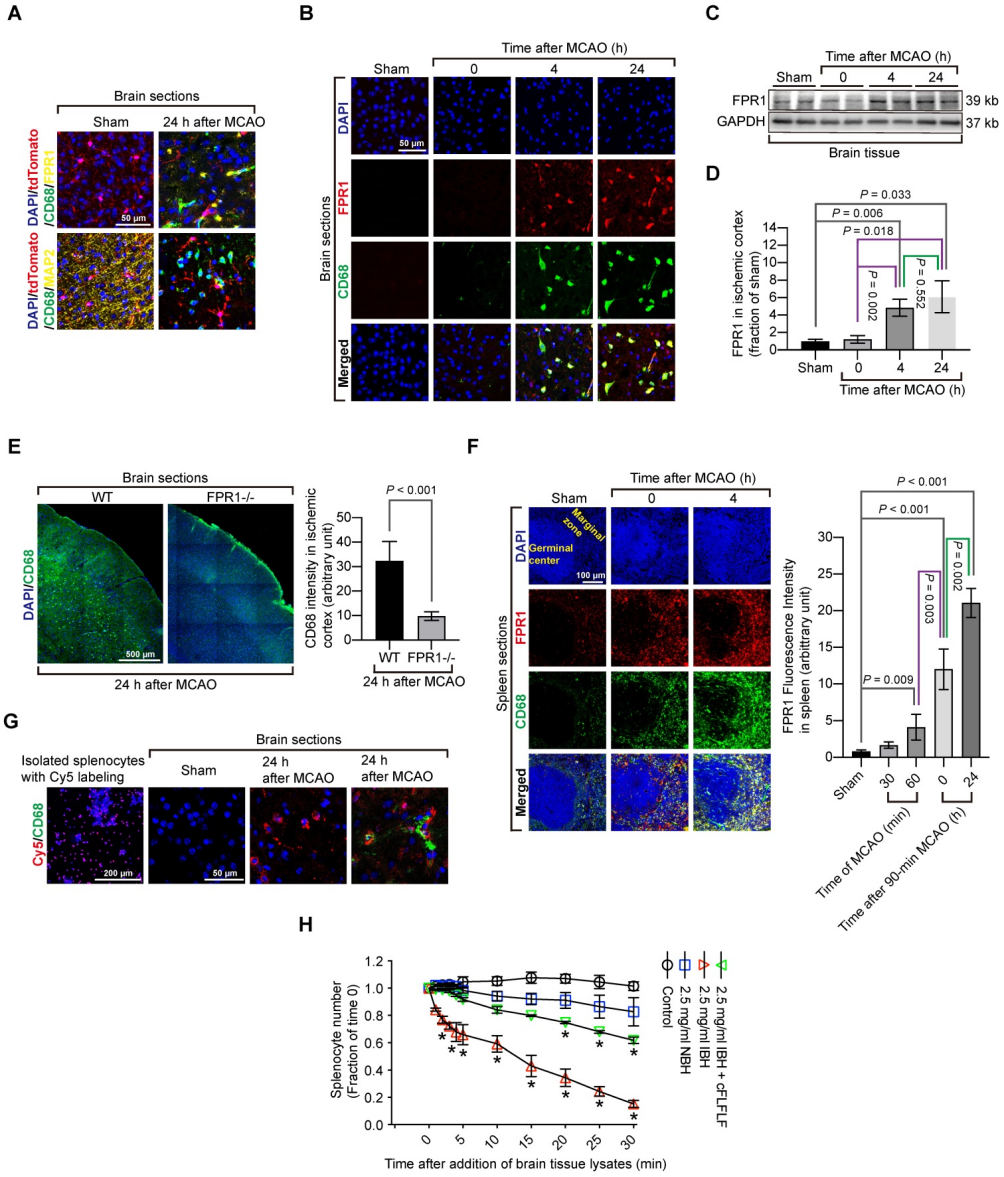
** Migration of splenocytes into the brain and the role of FPR1 in this migration.** Mice had 1.5 h MCAO and then various durations of reperfusion. **A:** immunostaining of brain sections harvested at 24 h after MCAO from the Cx3cr1CreER2 and Rosa26-LSL-tdTomato mice. Microglia in these mice have endogenous red fluorescence. Scale bar = 50 µm. **B:** immunostaining of brain sections harvested at various durations after MCAO from wild-type mice. Scale bar = 50 µm. **C:** representative images of Western blotting of FPR1 in the brain. **D:** quantification of FPR1 in the brain by Western blotting analysis.** E:** images of tile scanning and quantification of CD68 immunostaining in the ischemic cortex of wild-type mice and mice with *fpr1*^-/-^. Scale bar = 500 µm in the left image panels. **F:** images and quantification of CD68 immunostaining in the spleen of wild-type mice with or without MCAO. Scale bar = 100 µm in the left image panels. **G:** fluorescent images of wild-type mice that had sham surgery or 1.5 h MCAO and received intravenous injection of Cy5-labeled splenocytes after they had splenectomy. Scale bar = 200 µm in the left panel and 50 µm in the other three panels. **H:** quantification data of chemotaxis assay with CD-1 mouse splenocytes. Results in panels D, E, F and H are mean ± S.E.M. (n = 6 - 8 for panel D, 6 for panel E, 4 for panes F and H). One-way analysis of variance followed by the Tukey test was used for analyzing results in panels D and F, t test was used for analyzing data in panel E and one-way and two-way repeated measures analysis of variance followed by Tukey test was used for analyzing data in panel H. E. * P < 0.05 compared with the values at time 0.

Considering the possibility that activated microglia could have an elevated level of CD68, CD68 positive cells might include activated residential microglia. To rule out this possibility, we took advantage of a genetic tool. Mice containing Cx3cr1CreER2 were crossed with Rosa26-LSL-tdTomato mice whose proliferative microglia had endogenous red fluorescence after tamoxifen induction. Consistent with the wild-type mouse results, cells that were positively stained for CD68 cells did not have red fluorescence, suggesting that proliferative microglia may not contribute to CD68 positive cell population. None of CD68 positive cells were positive for MAP2 in these transgenic mice. Also, FPR1 staining did not appear in the microglial cells (Figure 3A). In wild-type mice, almost all CD68 positive cells in the ischemic brain tissues were positively stained with FPR1 (Figure 3B). Similar to the change of CD68 positive cells, the expression of FPR1 in the ischemic brain tissues was increased with time (Figure 3C-D). The intensity of CD68 positive staining in the ischemic brain tissues was much decreased in the *fpr1*^-/-^ mice (Figure 3E). These results suggest that peripheral cells migrated into the brain are FPR1 positive cells. This finding, along with the results that cFLFLF decreased CD68 positive cells into the brain, indicate that FPR1 is important for the migration of peripheral monocytes and macrophages into the brain.

Since the spleen contains a large number of immune cells and was labeled by Cy7-cFLFLF after brain ischemia, we determined whether splenocytes migrated into the ischemic brain tissues. There were a few CD68 positive cells in the spleen of sham operated mice. The majority of these cells were FPR1 positive. Cells that are positively stained for both CD68 and FPR1 in the spleen of a mouse with brain ischemia were increased dramatically after reperfusion (Figure 3F). Freshly isolated splenocytes were labeled *in vitro* with Cy5 and then injected intravenously into another mouse that had already had a splenectomy. There was no Cy5 labeled cells in the brain of sham operated mice but Cy5 labeled cells were in the ischemic brain tissues. Many of these cells were also positive for CD68 staining (Figure 3G). These results provide direct evidence that *ex vivo* labeled splenocytes migrate into ischemic brain tissues. Interestingly, *in vitro* chemotaxis assay showed that splenocytes did not migrate toward saline or normal brain tissue lysates [F(1,6) = 4.613, P = 0.075, comparing normal brain tissue lysate group with saline group] but were attracted by ischemic brain tissues [F(1,6) = 109.349, P < 0.001, comparing ischemic brain tissue lysate group with saline group] (Figure 3H), suggesting that ischemic brain tissues contain chemoattractants. This chemoattraction movement was significantly attenuated by cFLFLF [F(1,6) = 53.816, P < 0.001, comparing ischemic brain tissue lysate group with ischemic brain tissue lysate plus cFLFLF group] (Figure 3H), suggesting that FPR1 is involved in chemotaxis of splenocytes into the brain.

### Spleen weight was changed with the evolution of stroke and reperfusion might be needed for peripheral cells to migrate into the ischemic brain

**Figure 4:**
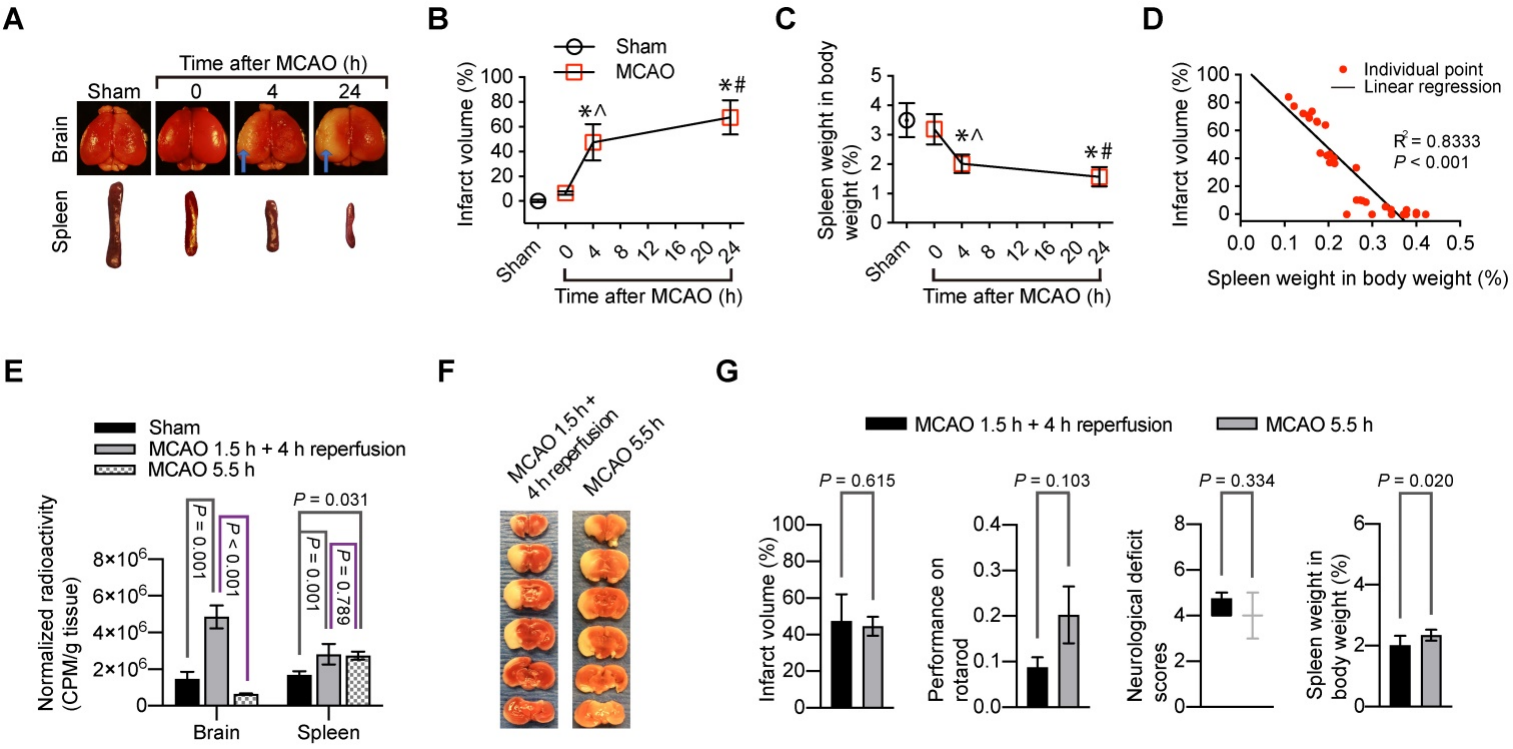
** Indications of the role of splenocyte migration into the brain in ischemic brain injury.** For panels A to D, mice had 1.5 h MCAO and then 4 h or 24 h reperfusion when the assessment was performed. **A:** representative photographs of brain and spleen. Blue arrows indicate infarct area. **B:** infarct volumes. **C:** spleen weights. **D:** correlation of brain infarct volumes and spleen weights. For panels E to G, mice were subjected to 1.5 h MCAO and then 4 h reperfusion or to 5.5 h MCAO. Their neurological function outcome was evaluated at the end of the experiments. Their brain and spleen were harvested immediately after the evaluation. **E:** Mice received ^99m^Tc-cFLFLF at ~37 MBq in 200 µl at the onset of reperfusion for mice that had 1.5 h MCAO plus 4 h reperfusion or 1.5 h after the onset of MCAO for mice that had 5.5 h MCAO. Their brain and spleen were harvested for counting their radioactivity *in vitro*. **F:** representative brain slices after TTC staining. **G:** neurological outcome and spleen weights. Results in panels B to E and G except for neurological deficit scores) are mean ± S.E.M. (n = 3 - 4 for panel E and = 8 for the other panels). Results of neurological deficit scores in panel G are in box plot. One-way analysis of variance followed by the Tukey test was used for analyzing results in panels B, C and E. Linear regression was used to analyze results in panel D. Results in panel G were analyzed by t-test or Mann-Whitney rank sum test. * P < 0.05 compared with the sham operated group. ^ P < 0.05 compared with values at time 0 (onset of reperfusion). # P < 0.05 compared with values at 4 h after MCAO.

The brain infarct volumes after MCAO were increased with time during the 24-h observation (Figure 4A-B). On the other hand, the spleen weight was decreased with time (Figure 4C). Interestingly, the brain infarct volume was correlated very well with the spleen weight (Figure 4D). These results suggest a critical role of the spleen in the evolution of brain infarction.

Since splenocytes were clearly migrated into ischemic brain tissues, we determined whether reperfusion was needed for the peripheral cells to migrate into the brain. ^99m^Tc-cFLFLF (37 MBq in 200 µl) was injected intravenously at the onset of reperfusion. These mice had 1.5 h MCAO and then 4 h reperfusion. Another group of mice had 5.5 h MCAO and received ^99m^Tc-cFLFLF at 1.5 h after the onset of MCAO. The brain and spleen of these groups of mice and sham operated mice that received ^99m^Tc-cFLFLF injection 4 h ago were harvested for quantification of radioactivity. As shown in Figure 4E, the radioactivity normalized by the body weight and brain weight in the mice with 1.5 h MCAO and then 4 h reperfusion was significantly higher than that in the sham operated mice and mice with 5.5 h MCAO. In fact, the normalized radioactivity in the brain of mice with 5.5 h MCAO was similar to that in the sham operated mice. On the other hand, the normalized radioactivity in the spleen of mice with 5.5 h MCAO or with 1.5 h MCAO and 4 h reperfusion was similar and was higher than that in the sham operated group. These results suggest that reperfusion is needed for peripheral cells to migrate into the brain (i.e., peripheral cells do not reach the ischemic brain tissues in the absence of blood flow in the acute phase). Interestingly, the infarct volumes tended to be smaller and neurological functions as assessed by neurological deficit scores and performance on rotarod tended to be better in the mice with 5.5 h MCAO than those mice with 1.5 h MCAO and 4 h reperfusion. However, these changes did not reach statistical significance (Figure 4F-G). Nevertheless, the spleen weight in the mice with 5.5 h MCAO was heavier than that in the mice with 1.5 h MCAO and 4 h reperfusion (Figure 4G). These results are consistent with the idea that reperfusion may be needed for peripheral cells to migrate into the brain. This migration may contribute to the reperfusion injury because the migration of peripheral inflammatory cells into the brain may harm the brain cells.

### cFLFLF induced neuroprotection and reduced neuroinflammation

**Figure 5:**
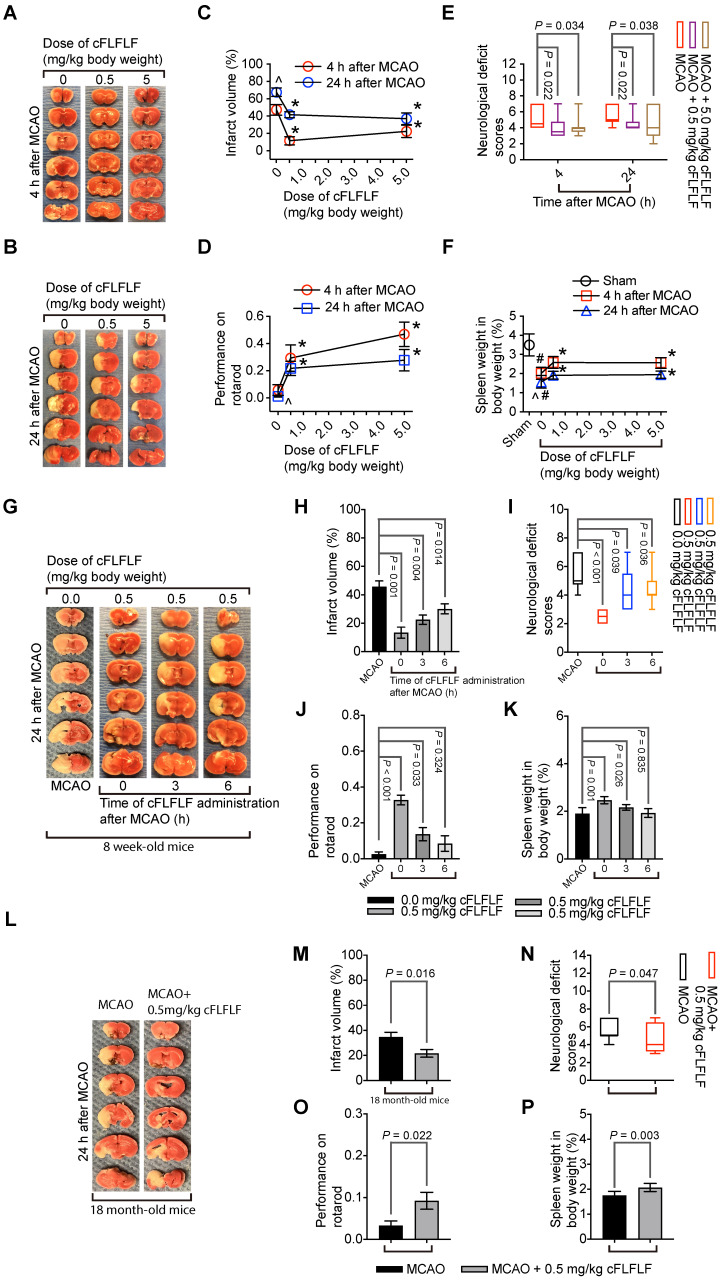
** Neuroprotective effects of cFLFLF.** CD-1 mice had 1.5 h MCAO. Their neurological outcome was evaluated 4 h or 24 h after MCAO. Panels A to F are results of the cFLFLF dose-response experiment with 4 or 24 h reperfusion time in young mice. **A** and **B:** representative brain slices after TTC staining. **C:** brain infarct volumes. **D:** performance on rotarod. **E:** neurological deficit score. **F:** spleen weight. Panels G to K are results of the cFLFLF protective time-window experiment with 24 h reperfusion time in young mice. **G:** representative brain slices after TTC staining. **H:** brain infarct volumes. **I:** neurological deficit scores **J**: performance on rotarod. **K:** spleen weight. Panels L to P are results of the cFLFLF protection experiment with 24 h reperfusion time in old mice. **L:** representative brain slices after TTC staining. **M:** brain infarct volumes. **N:** neurological deficit scores. **O:** performance on rotarod. **P:** spleen weight. Results in panels C, D, F, H, J, K, M, O and P are mean ± S.E.M. (n = 8 for panels C and F, 9 - 12 for 4 h reperfusion time groups in panel D, 10 to 14 for 24 h reperfusion time groups in panels D, 6 to 7 in panels H and K, 6 - 10 for panel J, 7 - 9 for panels M and P, 12 for panel O) and were analyzed by one-way analysis of variance followed by the Tukey test (panels C, D, F, H, K and J) or t-test (panel M, O and P). Results in panels E, I and N are in box plot (n = 9 - 12 for 4 h reperfusion time groups in panel E, 10 to 14 for 24 h reperfusion time groups in panel E, 6 - 10 for panel I, 12 for panel N) and were analyzed by one-way analysis of variance on ranks followed by the Tukey test (panels E and I) or Mann-Whitney rank sum test (panel N). ^ P < 0.05 compared with data of mice with 4 h reperfusion. * P < 0.05 compared with the corresponding values of mice without cFLFLF treatment. # P < 0.05 compared with the values of sham operated mice.

Since cFLFLF inhibited the migration of peripheral inflammatory cells into the brain, cFLFLF might be neuroprotective. Consistent with this idea, cFLFLF at 0.5 mg/kg (~18.4 nanomoles per mouse) or 5 mg/kg (~0.18 micromoles per mouse) given at the onset of reperfusion reduced brain infarct volume, decreased neurological deficit scores and improved performance on rotarod of mice with 1.5 h MCAO and either 4 h or 24 h reperfusion in 8-week old mice. The spleen weight of these mice with brain ischemia was persevered by cFLFLF. Of note, 0.5 mg/kg cFLFLF had already maximized the protective effects (Figure 5A-F). In this study, 6 out of 14 and 4 out of 12 mice died in the 0 mg/kg group, 2 out of 10 and 2 out of 10 mice died in the 0.5 mg/kg group, 1 out of 9 and 3 out of 11 mice died in the 5 mg/kg cFLFLF group within the 4 or 24 h reperfusion time, respectively. Their neurological deficit scores (assigned a score of 7) and performance on rotarod (assigned a value of 0) were included in the final data analysis but these mice did not contribute infarct volume data for the final analysis as we did before 19.

Similar to the finding described above, 0.5 mg/kg cFLFLF given at the onset of reperfusion in another experiment reduced brain infarct volume, improved neurological functions and increased the splenic weights assessed 24 h after brain ischemia in 8-week old mice. This dose of cFLFLF given at 3 h after the onset of reperfusion also achieved this protection. The same dose of cFLFLF given at 6 h after the onset of reperfusion reduced the brain infarct volume and improved neurological deficit scores but the improvement of performance on rotarod and the increase of the splenic weights were not statistically significant by this application (Figure 5G-K). In this study, 3 out of 10, 0 out of 6, 2 out of 9 and 1 out of 7 mice died in the groups of MCAO only, cFLFLF given at 0, 3 or 6 h after the onset of reperfusion, respectively, within 24 h reperfusion time. These results suggest that a delayed application of cFLFLF is neuroprotective.

Importantly, 0.5 mg/kg cFLFLF given at the onset of reperfusion reduced brain infarct volume, improved neurological functions and increased the splenic weights assessed 24 h after brain ischemia in 18-month old mice (Figure 5L-P). Five out of 12 and three out of 12 mice died in the 0 and 0.5 mg/kg cFLFLF groups, respectively, within 24 h reperfusion time. These results suggest that cFLFLF is protective in old mice.

**Figure 6:**
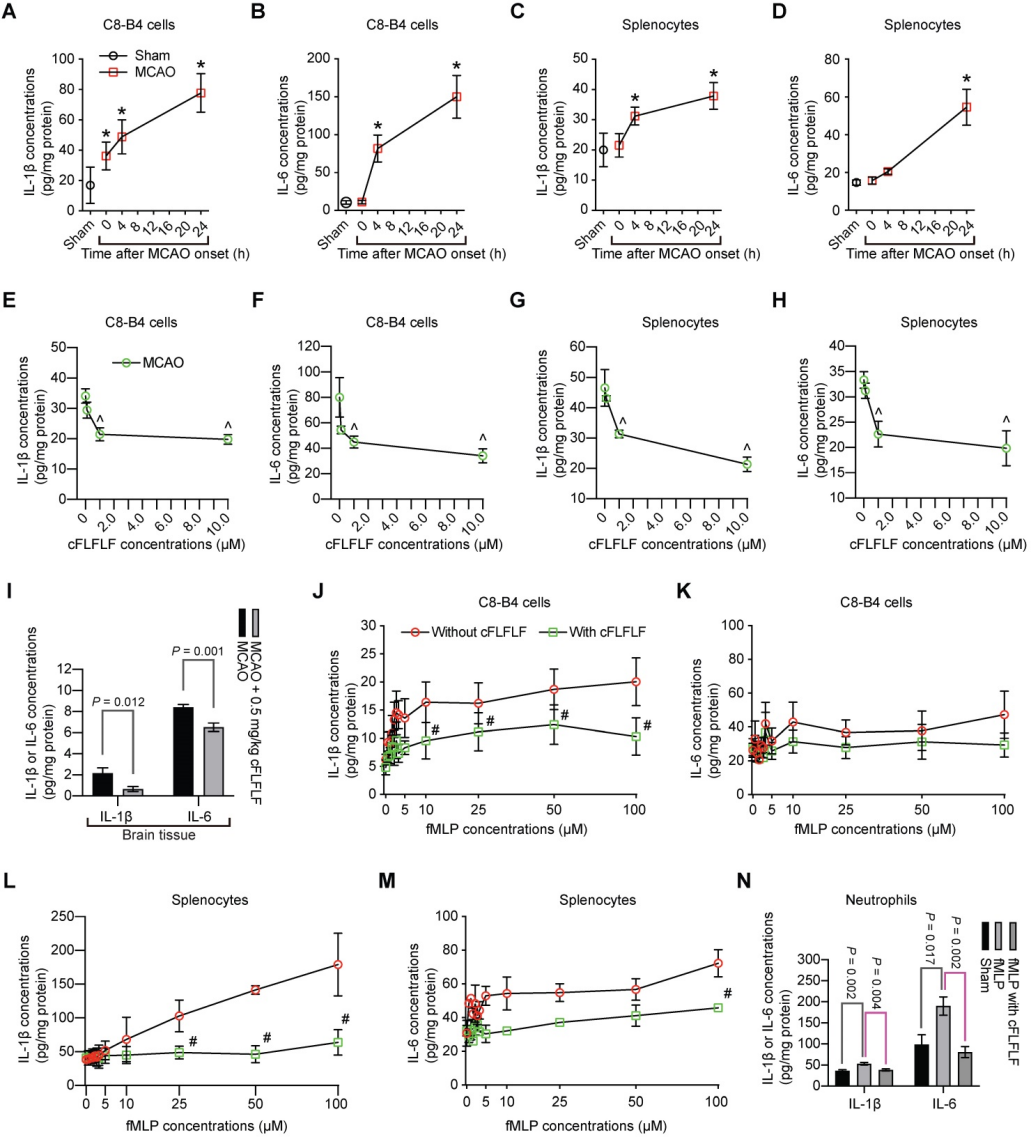
** Inhibition of proinflammatory cytokine production by cFLFLF. A to D:** IL-1β and IL-6 production after incubation with ischemic brain tissue lysates (IBH, 0.5 mg/well) prepared at various times after 1.5 h MCAO. **E to H:** IL-1β and IL-6 production after incubation with ischemic brain tissue lysates (0.5 mg/well) prepared at 4 h after 1.5 h MCAO. **I:** IL-1β and IL-6 expression in the left cerebral hemisphere harvested 4 h after 1.5 h MCAO. Mice received intravenous 0.5 mg/kg cFLFLF at the onset of reperfusion. For panels J to M, cells were incubated with various concentrations of fMLP in the presence or absence of 1 µM cFLFLF for 4 h. **J:** IL-1β from C8-B4 cells. **K:** IL-6 from C8-B4 cells. **L:** IL-1β from freshly isolated splenocytes. **M:** IL-6 from freshly isolated splenocytes. **N:** IL-6 and IL-1β from freshly isolated neutrophils in the presence or absence of 100 µM fMLP or 1.0 µM cFLFLF. Results are mean ± S.E.M. (n = 6 for panels A to H and N, 10 for panel I, 4 for panels J to M). Results were analyzed by one-way analysis of variance followed by the Tukey test for panels A to H and N, t-test for panel I and two-way repeated measures analysis of variance followed by Tukey test for panels J to M. * P < 0.05 compared with the sham operated mice. ^ P < 0.05 compared with data without cFLFLF treatment. # P < 0.05 compared with the corresponding values of no cFLFLF group.

Interestingly, in addition to inducing splenocyte migration, ischemic brain tissue lysates increased the production of IL-1β and IL-6 from C8-B4 cells, a microglial cell line 25, or from freshly isolated splenocytes. These increases were dose-dependently inhibited by cFLFLF (Figure 6A-H). Similar to these *in vitro* data, cFLFLF inhibited the expression of IL-1β and IL-6 in the ischemic brain tissues (Figure 6I).

To determine whether the effects of cFLFLF on inflammatory cytokine production were through FPRs, we incubated C8-B4 cells or freshly prepared splenocytes with fMLP, an agonist of FPRs 11. fMLP dose-dependently increased IL-1β and had much less effect on IL-6. The effects on the production of these cytokines in the freshly isolated splenocytes were more robust compared to those in the C8-B4 cells. cFLFLF at 1 µM attenuated the effects of fMLP on IL-1β [F(1,6) = 35.254, P = 0.001] and IL-6 [F(1,6) = 42.05, P < 0.001] in the splenocytes (Figure 6J-M), suggesting that the cFLFLF-induced inhibition of proinflammatory cytokine production was through its effects on FPRs in the splenocytes. The ineffectiveness of cFLFLF on IL-1β [F(1,6) = 1.910, P = 0.216] and IL-6 [F(1,6) = 0.366, P = 0.568] in the C8-B4 cells may be because fMLP was not an effective stimulator in these cells. Since neutrophils migrated into the ischemic brain tissues as shown in a previous section, the effects of cFLFLF on the production of proinflammatory cytokines in the neutrophils were determined. Similar to the results of C8-B4 cells and splenocytes, fMLP increased IL-6 and IL-1β and cFLFLF blocked this increase in the neutrophils (Figure 6N). These results suggest that cFLFLF inhibits FPR1-dependent production of inflammatory cytokines in the neutrophils.

### FPR1 affected brain ischemic tolerance and was necessary for the inhibitory effects of cFLFLF on proinflammatory cytokine production in the spleen and brain after brain ischemia

**Figure 7:**
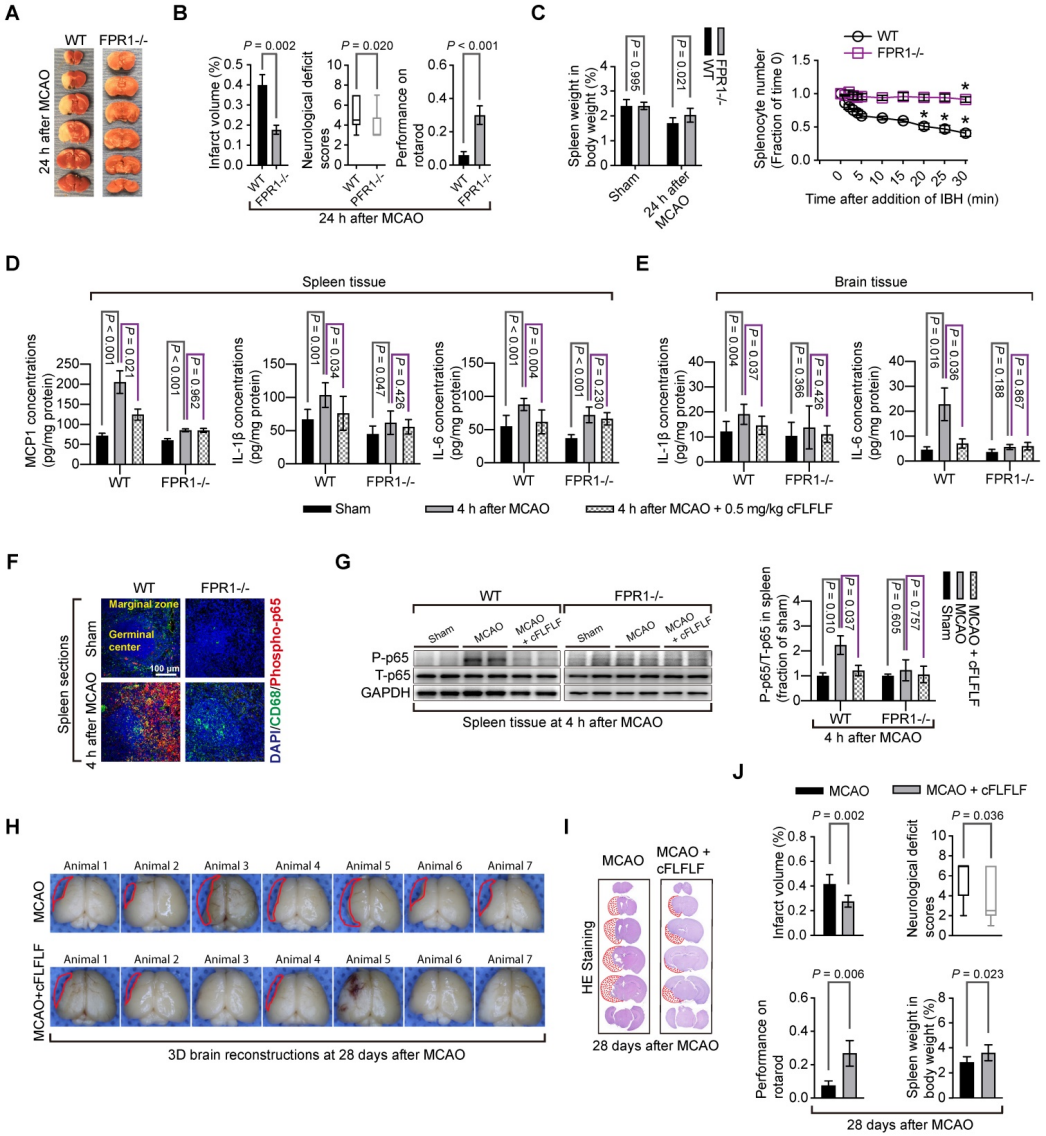
** Role of FPR1 in neuroinflammation and ischemic brain tolerance.** For panels A and B, wild-type (WT) mice and *fpr1*^-/-^ mice had 1.5 h MCAO and then 24 h reperfusion. **A**: representative brain slices after TTC staining. **B**: neurological outcome. **C**: left panel: spleen weight at baseline and after 1.5 h MCAO and 24 h reperfusion, right panel: quantification data of *in vitro* chemotaxis assay of splenocytes. Ischemic brain tissues for inducing chemotaxis movement of splenocytes were prepared from wild-type mice at 24 h after MCAO. * P < 0.05 compared with the values at time 0. For panels D to G, wild-type mice and *fpr1*^-/-^ mice had 1.5 h MCAO and then 4 h reperfusion. Their brain and spleen were harvested for assessment. **D:** cytokine production from the spleen. **E:** cytokine production from the brain. **F:** immunostaining of spleen section. **G:** Western blotting analysis of phospho-p65 (P-p65) from spleen tissues. For treatment, 0.5 mg/kg cFLFLF was administrated intravenously at the onset of reperfusion. For panels H to J: wild-type mice were subjected to 1.5 h MCAO and the neurological outcome and spleen weight were assessed 28 days after the MCAO. Three doses of cFLFLF at 5 mg/kg were administrated intravenously with the first dose at the onset of reperfusion and then every 24 h. **H:** 3D reconstruction images of the brain. **I:** representatives of 2D images of brain sections with hematoxylin and eosin staining. **J:** quantification of neurological outcome parameters and spleen weight. Results in panels B, C, D, E, G and J are mean ± S.E.M. (n = 9 to 14 for performance on rotarod in panel B, 4 for chemotaxis assay data in panel C, 6 for panel G, 7 for infarct volume and spleen weight, and 12 to 23 for performance on rotarod in panel J, 8 for all other panels). Results of neurological deficit scores in panels B (n = 9 to 14) and J (n = 12 to 23) are in box plot and were analyzed by Mann-Whitney rank sum test. The other results in panels B and J and the left panel of panel C were analyzed by t-test. The right panel of panel C was analyzed by one-way and two-way repeated measures analysis of variance followed by Tukey test. Results in panels D, E and G were analyzed by one-way analysis of variance followed by the Tukey test.

Mice with* fpr1*^-/-^ had reduced brain infarct volume, decreased neurological deficit scores and improved performance on rotarod compared with wild-type mice after a 1.5 h MCAO and 24 h reperfusion (Figure 7A-B). In this study, 6 out of 14 wild-type mice and 1 out of 9 *fpr1*^-/-^ mice died during the 24 h reperfusion time. The *fpr1*^-/-^ mice had less spleen weight decrease than wild-type mice. Consistent with this result, very few of their splenocytes moved toward brain lysates in the *in vitro* chemotaxis assay (Figure 7C). FPR1 gene integrity was a significant factor to affect splenocyte migration toward brain lysates [F(1,6) = 316.240, P < 0.001].

There was an increase in MCP1, IL-1β and IL-6 in the spleen of wild-type mice and *fpr1*^-/-^ mice and IL-1β and IL-6 in the brain of wild-type mice after brain ischemia. cFLFLF inhibited these increases in the wild type mice but not in the *fpr1*^-/-^ mice (Figure 7D-E). This pattern of changes also occurred with the fraction of phospho-p65, an active sub-unit of NF-κB, in the total p65 (Figure 7F-G). These results suggest that the inhibitory effects of cFLFLF on proinflammatory cytokine production are mediated by FPR1.

### cFLFLF improved long-term neurological outcome after brain ischemia

To determine whether cFLFLF-induced neuroprotection was long-lasting, neurological outcome was evaluated 28 days after brain ischemia. Mice treated with cFLFLF had reduced brain infarct volume, decreased neurological deficit scores and improved performance on rotarod compared with mice without the treatment (Figure 7H-J). In this study, 16 out of 23 mice and 5 out of 12 mice in the groups that received 0 or 5 mg/kg cFLFLF, respectively, died during the observation period of 28 days (P = 0.217). Interestingly, the spleen weights of mice treated with cFLFLF were heavier than those of mice without cFLFLF treatment even in this late phase after brain ischemia (Figure 7J).

## Discussion

The contribution of the spleen to ischemic brain injury has been indicated because splenectomy before brain ischemia attenuates ischemic brain injury 27. Indirect evidence has suggested that splenocytes migrate into ischemic brain tissues because splenectomy or irradiation of the spleen reduces the number of peripheral cells in these tissues. However, their role in neuroinflammation after brain ischemia is not well defined. Our study confirmed that ischemic stroke reduced spleen weight and that this reduction is evolving after the stroke, a situation that is similar to that in humans reported recently 8. In addition, we found that the spleen weight reduction correlates very well with the brain infarct volume, consistent with the finding from a previous study 37. More importantly, we provide direct evidence that splenocytes migrate into the brain because cells positively stained for CD68, a marker for monocytes and macrophages 32, or Ly6G, a marker for neutrophils 34, that were not microglia appeared in the brain and splenocytes labeled with Cy5 were seen in the brain. Thus, splenocytes may not only contribute to the systemic inflammatory response but also neuroinflammation, indicating a direct effect on ischemic brain tissues.

Brain ischemia and reperfusion induced the presence of a large number of CD68 positive cells in the brain. Most of these cells are not positively stained for TMEM 119 or Iba-1, two specific microglial markers 35. In addition, these CD 68 positive cells are not co-labeled with red fluorescent dye that was endogenously expressed in microglial cells of the Rosa26-LSL-tdTomato mice. These results suggest that these cells are peripheral monocytes and macrophages that migrate into the brain. In supporting this suggestion, these cells are co-labeled with FPR1 and FPR1 staining did not appear in the microglial cells. Thus, similar to CD68, FPR1 may be mostly expressed in the peripheral monocytes and macrophages and can be used as a marker for these cells. Interestingly, ischemic brain tissue lysates but not the normal brain tissue lysates significantly increased the migration of splenocytes. cFLFLF attenuated the migration of splenocytes *in vitro* and the CD68 positive cells into the brain. These results suggest a significant role of FPR1 in the migration of peripheral inflammatory cells into ischemic brain tissues. Interestingly, human peripheral blood mononuclear cells harvested 24 h after the onset of ischemic stroke have an increased expression of FPR1-like gene 38, suggesting that a similar process may occur in humans.

Our results indicate that reperfusion may be necessary for cells to migrate into ischemic brain tissues. Cy7-cFLFLF did not light up the brain immediately after the brain ischemia, unlike the situation in the spleen. Since the inflammatory focus in the brain was clearly identified by Cy7-cFLFLF 22 min after the brain ischemia when Cy7-cFLFLF was injected immediately at the onset of reperfusion, we subjected animals to a 5.5 h MCAO and injected ^99m^Tc-cFLFLF to the animals 1.5 h after the onset of MCAO. The brain and spleen were harvested 5.5 h after the onset of MCAO for measuring the radioactivty in the organs. The amount of ^99m^Tc-cFLFLF in the brain of these animals was similar to that of sham operated animals and was much lower than the animals that received 1.5 h MCAO and 4 h reperfusion, while the amount of ^99m^Tc-cFLFLF in the spleen of animals with 5.5 h MCAO was similar to that of animals with 1.5 h MCAO and 4 h reperfusion and was higher than that of sham operated animals. In addition, the spleen weight of 5.5 h MCAO mice was heavier than that of mice with 1.5 h MCAO and 4 h reperfusion. These results suggest that the reperfusion is needed for inflammatory cells to migrate into the ischemic brain tissues. This suggestion is concerivable because re-establishing blood flow shall be needed to bring the cells into these tissues. Consistent with this possibility, although the splenic weights are decreased transiently in rats with permanent MCAO by an intravascular suture method, the labeled splenic cells appear in the blood and remain in the blood vessels of the brain for the duration of the oberservation time up to 96 h after the onset of brain ischemia 39. Interestingly, the neurological outcome of mice with 5.5 h MCAO tended to be better than the mice with 1.5 h MCAO and 4 h reperfusion. These results suggest a damaging role of reperfusion in brain injury after brain ischemia. Consistent with this observation, the longer the reperfusion time, the more powerful the brain tissue lysates to induce proinflammatory cytokine production.

An important finding of our study is that cFLFLF is neuroprotective. Application of cFLFLF reduced brain infarct volume and improved neurological functions whether the assessment was performed in the acute phase (24 h) or a delayed phase (28 days) after brain ischemia in 8-week old mice. The protection remains when there is a delay (up to 3 to 6 h after the onset of reperfusion) in the administration of cFLFLF. Similarly, cFLFLF provides neuroprotection in old mice. Also, cFLFLF decreased ischemic stroke-induced spleen weight loss and reduced splenocyte migration *in vitro* and migration of peripheral cells into the brain. Finally, cFLFLF inhibited the production of proinflammatory cytokines in the brain and spleen after brain ischemia and from C8-B4 cells or splenocytes stimulated by ischemic brain tissue lysates or fMLP. These results provide initial and strong evidence that cFLFLF inhibits brain injury and neuroinflammation after brain ischemia and reperfusion. These effects may be at least partly due to the decrease of migration of peripheral cells including splenocytes into the brain by cFLFLF.

Another important finding of our study is that FPR1 plays a critical role in mediating the migration of peripheral monocytes and neutrophils into ischemic brain tissues and altering the degree of ischemic brain injury. cFLFLF, an antagonist for FPR1, reduced the migration of the peripheral cells into the brain and improved neurological outcome after brain ischemia. Our results also suggest that cFLFLF mainly binds to FPR1 because cy5-cFLFLF had very little binding to splenocytes of *fpr1*^-/-^ mice compared with cells from wild-type mice and that cFLFLF attenuated the production of proinflammatory cytokines in the spleen of wild type mice but not in the spleen of *fpr1*^-/-^. Mice with *fpr1*^-/-^ had fewer peripheral cells migrating into ischemic brain tissues. These *fpr1*^-/-^ mice had better neurological outcomes and less spleen weight loss than wild-type mice after brain ischemia. Although brain ischemia and reperfusion increased proinflammatory cytokine production in the spleen of both wild-type and *fpr1*^-/-^ mice, this increased cytokine production occurred in the ischemic brain tissues of wild-type mice but not *fpr1*^-/-^ mice. Together, these results suggest that FPR1 is a novel molecular target for regulating the recruitment and migration of peripheral monocytes, macrophages and neutrophils into the ischemic brain tissues and for attenuating neuroinflammation to provide neuroprotection. These results further suggest a critical role of migrated peripheral monocytes, macrophages and neutrophils in ischemic stroke-induced neuroinflammation and brain injury.

Our study showed that there were Ly6G positively stained cells in the ischemic brain tissues and that there was an increase in Ly6G proteins in these tissues after reperfusion. These results suggest that peripheral neutrophils infiltrate into the ischemic brain tissues. Neutrophils may have a detrimental effect during acute phase after brain ischemia because of their proinflammatory and pro-oxidative properties 40, 41. Consistent with this effect, neutrophiles have a FPR1-dependent increase of proinflammatory cytokine production. The migration of neutrophils into the brain was FPR1-dependent and blocking FPR1-dependent cell migration was neuroprotective in our study. However, neutrophils can help remove cell debris and are involved in maintaining an immunological defense 40, 41. Also, FPR-dependent cell migration into the injured tissues may be important for wound healing because double knockout of *fpr1* and *fpr2* delay wound healing 42. Similarly, FPR1 is involved in angiogenesis, cell proliferation and neuronal differentiation 43, 44. Thus, careful regulation of neutrophil infiltration and FPR1 functions is needed to reduce their detrimental effect and preserve the beneficial roles of these cells during peri-brain ischemic period.

It is known that FPR1 signaling can lead to NF-κB activation 45. Consistent with this knowledge, phospho-p65 was increased in the spleen of wild-type mice and this increase was attenuated by cFLFLF. There was no increase of phospho-p65 in the spleen of *fpr1^-/-^* mice. Since NF-κB is a critical molecule to regulate the expression of proinflammatory cytokines and chemokines 3, 25, it is possible that NF-κB is an upstream molecule targeted by cFLFLF for its effects on MCP1, IL-1β and IL-6 in the spleen and ischemic brain tissues 3, 25, 46.

Our findings have significant implications. FPR1 may be proved to be a reasonable target to induce neuroprotection in humans in future studies because FPR1 is expressed in activated cells 9 and blocking FPR1 may not affect baseline functions of monocytes and macrophages, a desirable situation for patients with stroke. Also, cFLFLF may be a suitable agent to specifically induce neuroprotection. Other agents that specifically inhibit the migration of peripheral monocytes and macrophages into ischemic brain tissues via working on FPR1 may be developed for neuroprotection. This line of research may be very useful in the context that more patients will receive therapies to establish blood flow to the ischemic brain tissues with the development of techniques and equipment for this purpose. Applying agents or interventions that reduce peripheral monocyte or macrophage migration into ischemic brain tissues may be a reasonable approach to provide neuroprotection. Our study has shown a good time window (up to 6 h after the onset of reperfusion) for cFLFLF to be neuroprotective. It may remain clinically relevant even if the agent has to be applied at the onset of reperfusion for it to be neuroprotective because an increased number of patients will be under our care when the reperfusion begins.

Our study has limitations. It appears that brain ischemia alone can activate splenocytes in our study. It is not known yet whether this effect is mediated by signaling molecules in the blood or through the nervous system. Future pre-clinical studies will define these unknowns.

**Figure 8:**
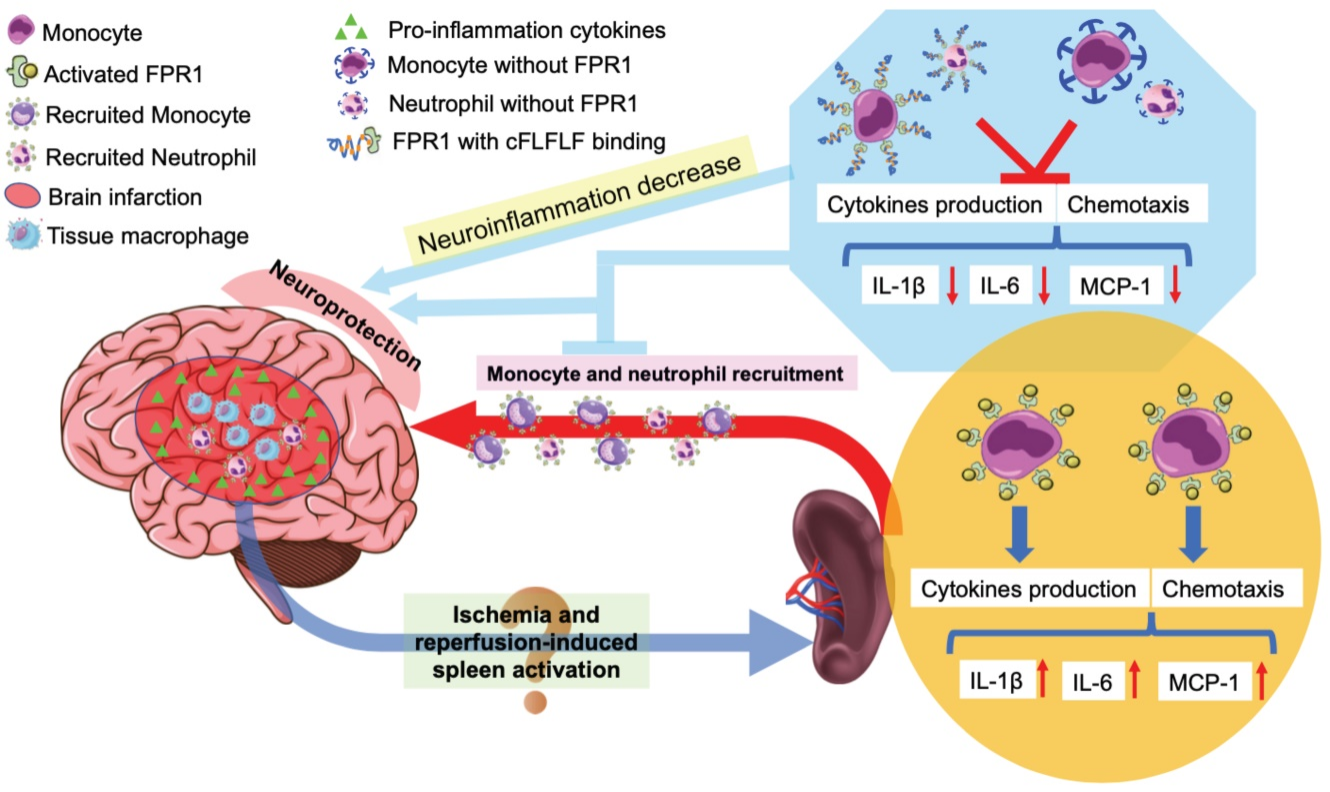
Schematic presentation of the role of splenocyte migration in ischemic brain injury and the role of FPR1 in this migration.

In summary, we have shown that reperfusion is needed for peripheral cells to migrate into the ischemic brain tissues. Splenocytes can migrate into the brain. cFLFLF inhibits splenocyte migration and neuroinflammation after brain ischemia and provides neuroprotection. These cFLFLF effects may be via antagonizing FPR1 (Figure 8). Our results also suggest that splenocytes have a direct effect on ischemic brain tissues to worsen ischemic brain injury.

## Highlights

- Splenocytes are activated after brain ischemia and migrate into ischemic brain tissues;
- Splenocyte activation and migration into the brain depend on formyl peptide receptor 1;
- Blocking formyl peptide receptor 1 reduces neuroinflammation after brain ischemia;
- Blocking formyl peptide receptor 1 improves long-term outcome after ischemic brain.

## Supplementary Material

Supplementary methods, figures.
