# Supplementary material for: Critical role of FPR1 in splenocyte migration into brain to worsen inflammation and ischemic brain injury in mice
Source: Theranostics. 2022 Mar 21;12(7):3024–44. doi: 10.7150/thno.57218 (PMC9065176; doi:10.7150/thno.57218)
Supplement: Supplementary file 1 — Supplementary methods, figures. [file thnov12p3024s1.pdf]

Supplemental materials

General Methods

Chemicals used for intermediates and peptide synthesis were procured from standard sources, such as Sigma-Aldrich (St. Louise, MO) and Acros Organics (New Jersey) and were used as is without further purification. The peptide synthesis was performed on CS Bio automated peptide synthesizer (Menlo Park, CA) using standard Fmoc peptide protocol. Fmoc protected amino acids were purchased from Anaspec (Fremont, CA) or Applied Biosystem (Foster City, CA). Ultrapure water (resistivity, 18.2 MΩ cm) used for making solutions was obtained from Milli-Q Direct Ultrapure Water System from Millipore (Billerica, MA, USA). High-performance liquid chromatography (HPLC) grade solvents were purchased from Fisher Scientific (Pittsburgh, PA, USA). Intermediates and final compounds were purified by preparative and semi-preparative reversed-phase HPLC using photodiode array detector (PDA) (Xiao et al., 2012).

Synthesis and purification of cFLFLF peptide

The synthetic peptide cinnamoyl-phenylalanyl-(D) leucine-phenylalanyl-(D) leucine-phenylalanine (cFLFLF) was synthesized using a standard solid phase Wang-resin supported Fmoc peptide synthesis protocol using an auto synthesizer (CS Bio Company), similar to a previous publication (Zhang et al., 2015). One gram of preloaded phenylalanyl Wang resin (0.33 mmol/g, Ana Spec, Inc.) was soaked with N, N-dimethylformamide (DMF) and sequentially Fmoc protected and coupled with D-leucine, phenylalanine, and D-leucine-phenylalanine. At the end of peptide Fmoc synthesis the terminal phenylalanine was deprotected and the amine was conjugated with cinnamic acid. The peptide was removed from solid phase upon treatment with 1% water containing trifluoroacetic acid (TFA, 2 mL). The crude peptide solution in TFA was concentrated under reduced pressure (~0.1 mL) and precipitated as a solid by adding to ice-water (5.0 g). This solid was filtered and vacuum dried (176 mg, 65%), and final purification was achieved with preparative HPLC, using a C18 column and a gradient from 60% acetonitrile:H_2O to 100% acetonitrile as eluent prior to confirmation of product by matrix-assisted laser desorption-time of flight (MALDI-TOF) mass spectrometry on a Bruker Microflex system (m/z 816) (Locke et al., 2009).

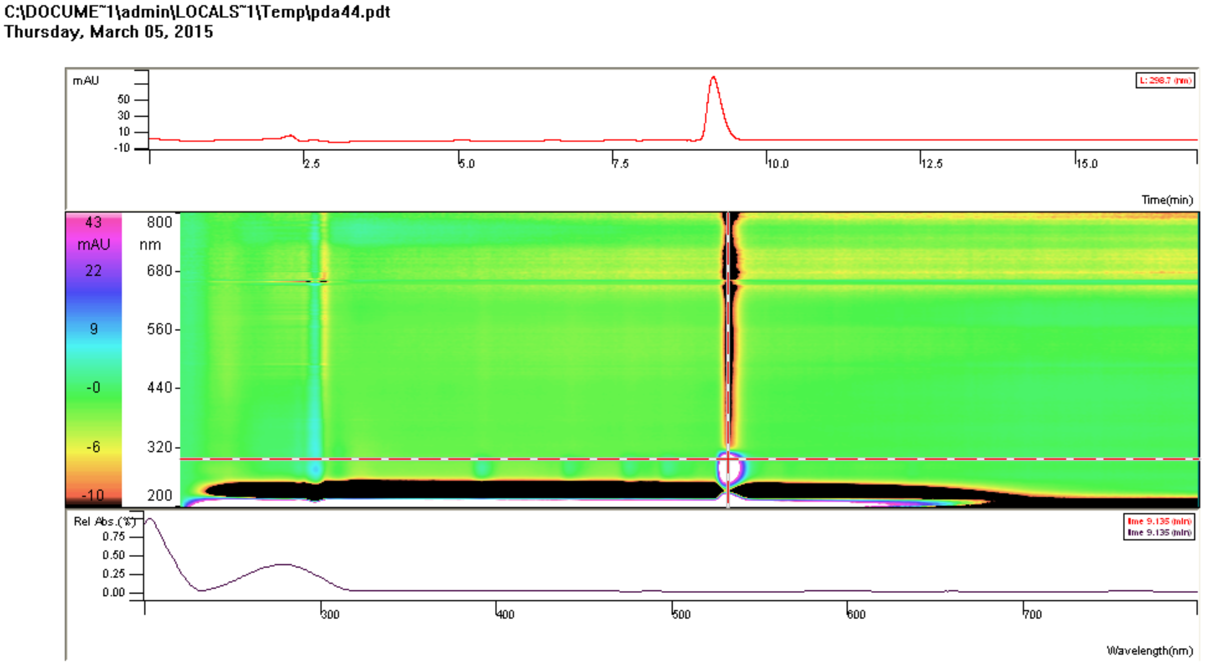

Supplemental Figure 1. Analytical HPLC chromatogram of cFLFLF using PDA detector.

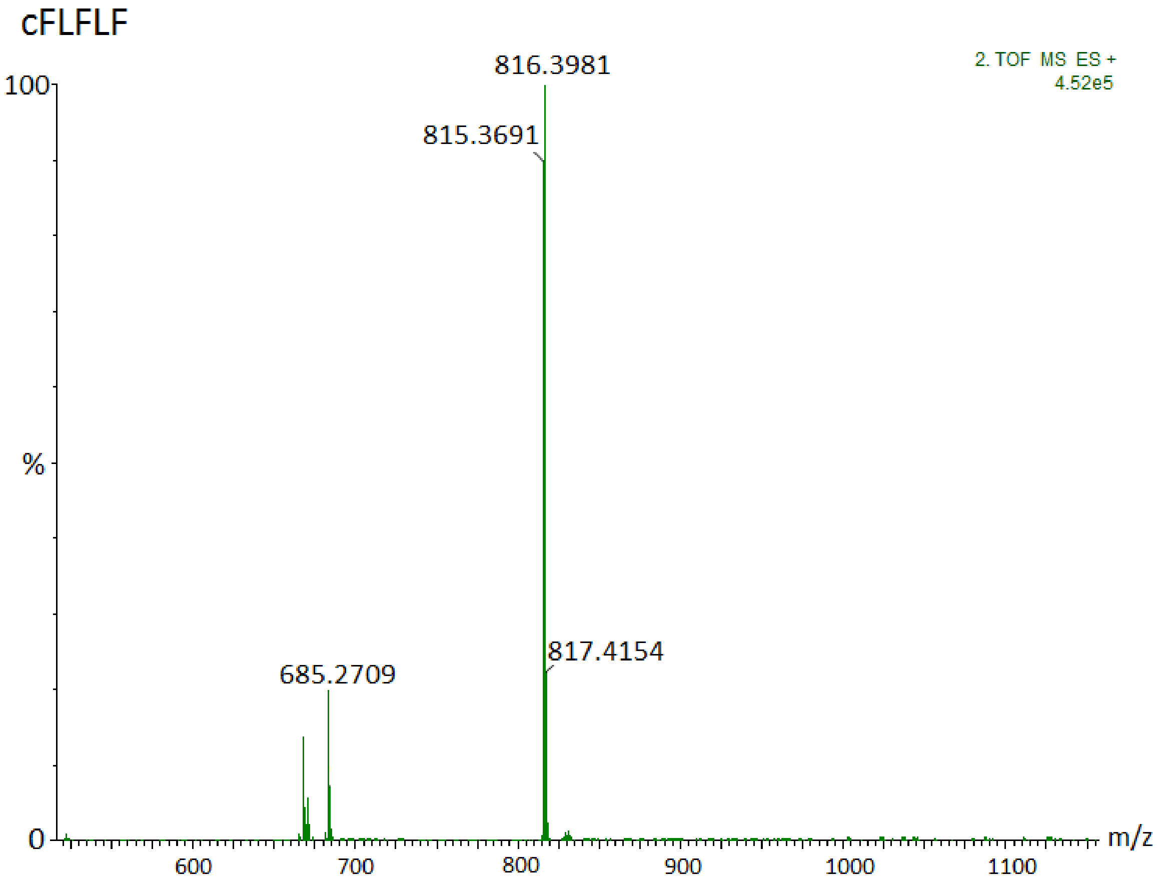

Synthesis and purification of fluorescence labeled probes cFLFLF-PEG-Cy7/Cy5

The detailed chemical synthesis and characterization of cFLFLF-PEG76-NH2 precursor is reported previously (Zhang et al., 2010) as shown in chemical scheme below. The cFLFLF-PEG76-NH3.OCOCF3 salt (5.0 mg, ~1.2 mM) obtained from deprotection of N-^{t}Boc protected intermediate was dissolved in acetonitrile/sodium borate buffer (0.1M, pH 8.5, 9:1, 2 mL) and chilled to 5 – 10^{0}C ice water bath. The addition to this chilled solution was the solution of either Cy7-NHS ester (GE Healthcare, Piscataway, NJ) or Cy5-NHS ester (GE HealthCare, Piscataway, NJ) (~1.0 mg, 5 - 7 fold excess) in 1 mL dry acetonitrile. The mixture was stirred for a few hours over which time the temperature of outside bath reach to room temperature. Analytical HPLC using Alltech's Apollo C18 5 µm column with PDA detector indicated formation of desired product. The solution was concentrated under reduced pressure and concentrated residue was purified to homogeneity (> 95%) with semi-prep RP HPLC chromatography. HPLC was performed on a Varian ProStar system (models: pumps, 210; column valve module, 500; fraction collector, 701) (Varian Instruments) equipped with an Alltech Apollo C18 semi-preparative column (5 µm, 250×10 mm) (Grace Davison Discovery Sciences) using PDA detector. Gradient mobile phases were used for HPLC: solvent A (0.1% TFA in water) and solvent B (0.1% TFA in 80% aqueous acetonitrile). The mobile phase gradient varied according to characteristics of compounds for 30 min at a flow rate 3 mL/min, fractions collected at λ=650 and λ=750 nm for cFLFLF-PEG$_{76}$-Cy5 and cFLFLF-PEG$_{76}$-Cy7, respectively.

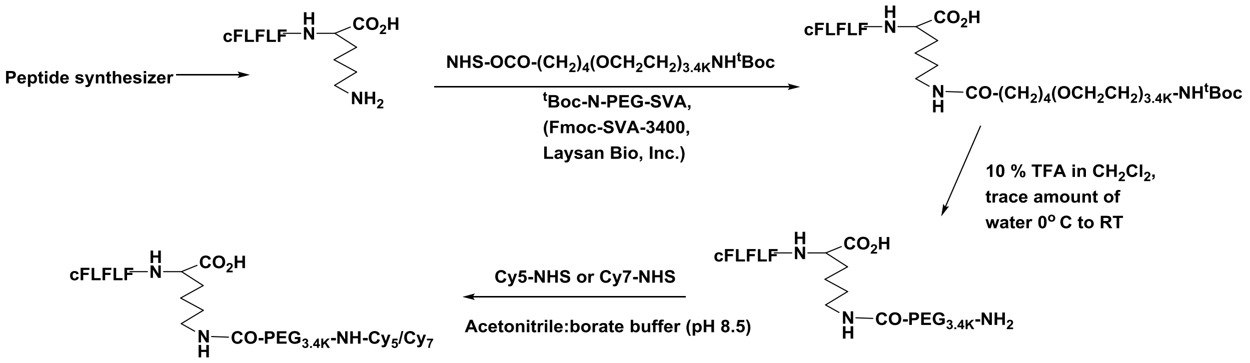

C:\DOCUME~1\admin\LOCALS~1\Temp\pda44.pdt
Thursday, February 06, 2014

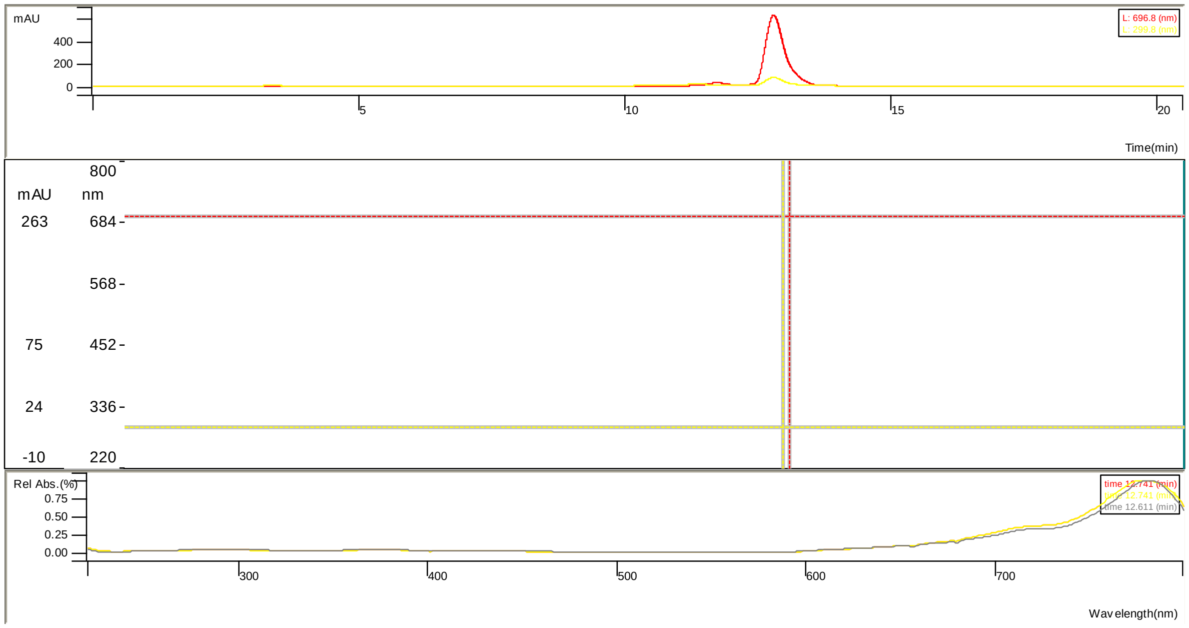

Supplemental Figure 2. Analytical HPLC chromatogram of cFLFLF-PEG-Cy7 using PDA detector

Synthesis of ^{99m}Tc-cFLFLF

The detailed synthesis of ^{99m}Tc-cFLFLF is reported previously (Charles et al., 2020). The cFLFLF-PEG-NH_2 was conjugated with HYNIC-NHS ester to afford cFLFLF-PEG-HYNIC, which was then metallated with ^{99m}Tc.

References:

Locke LW, Chordia MD, Zhang Y, Kundu B, Kennedy D, Landseadel J, Xiao L, Fairchild KD, Berr SS, Linden J, Pan D (2009) A novel neutrophil-specific PET imaging agent: cFLFLFK-PEG-64Cu. J Nucl Med 50:790-797.

Xiao L, Zhang Y, Berr SS, Chordia MD, Pramoonjago P, Pu L, Pan D (2012) A novel near-infrared fluorescence imaging probe for in vivo neutrophil tracking. Mol Imaging 11:372-382.

Zhang Y, Kundu B, Zhong M, Huang T, Li J, Chordia MD, Chen MH, Pan D, He J, Shi W (2015) PET imaging detection of macrophages with a formyl peptide receptor antagonist. Nucl Med Biol 42:381-386.

Zhang Y, Xiao L, Chordia MD, Locke LW, Williams MB, Berr SS, Pan D (2010) Neutrophil targeting heterobivalent SPECT imaging probe: cFLFLF-PEG-TKPPR-99mTc. Bioconjug Chem 21:1788-1793.

Charles EJ, Chordia MD, Zhao Y, Zhang Y, Mehaffy JH, Glover DK, et al. (2020) SPECT imaging of lung ischemia-reperfusion injury using [99mTc]cFLFLF for molecular targeting of formyl peptide receptor 1. Am J Physiol Lung Cell Mol Physiol 318: L304–L313.
